# 5-methylcytosine promotes mRNA export — NSUN2 as the methyltransferase and ALYREF as an m^5^C reader

**DOI:** 10.1038/cr.2017.55

**Published:** 2017-04-18

**Authors:** Xin Yang, Ying Yang, Bao-Fa Sun, Yu-Sheng Chen, Jia-Wei Xu, Wei-Yi Lai, Ang Li, Xing Wang, Devi Prasad Bhattarai, Wen Xiao, Hui-Ying Sun, Qin Zhu, Hai-Li Ma, Samir Adhikari, Min Sun, Ya-Juan Hao, Bing Zhang, Chun-Min Huang, Niu Huang, Gui-Bin Jiang, Yong-Liang Zhao, Hai-Lin Wang, Ying-Pu Sun, Yun-Gui Yang

**Affiliations:** 1 Center for Reproductive Medicine, The First Affiliated Hospital of Zhengzhou University, Zhengzhou, Henan 450000, China; 2 Key Laboratory of Genomic and Precision Medicine, Collaborative Innovation Center of Genetics and Development, Beijing Institute of Genomics, Chinese Academy of Sciences, Beijing 100101, China; 3 School of Life Science, University of Chinese Academy of Sciences, Beijing 100049, China; 4 State Key Laboratory of Environmental Chemistry and Ecotoxicology, Research Center for Eco-Environmental Sciences, Chinese Academy of Sciences, Beijing 100085, China; 5 Sino-Danish College, University of Chinese Academy of Sciences, Beijing 100049, China; 6 National Institute of Biological Sciences, Beijing 102206, China

**Keywords:** m^5^C, mRNA export, ALYREF, NSUN2

## Abstract

5-methylcytosine (m^5^C) is a post-transcriptional RNA modification identified in both stable and highly abundant tRNAs and rRNAs, and in mRNAs. However, its regulatory role in mRNA metabolism is still largely unknown. Here, we reveal that m^5^C modiﬁcation is enriched in CG-rich regions and in regions immediately downstream of translation initiation sites and has conserved, tissue-specific and dynamic features across mammalian transcriptomes. Moreover, m^5^C formation in mRNAs is mainly catalyzed by the RNA methyltransferase NSUN2, and m^5^C is specifically recognized by the mRNA export adaptor ALYREF as shown by *in vitro* and *in vivo* studies. NSUN2 modulates ALYREF's nuclear-cytoplasmic shuttling, RNA-binding affinity and associated mRNA export. Dysregulation of ALYREF-mediated mRNA export upon NSUN2 depletion could be restored by reconstitution of wild-type but not methyltransferase-defective NSUN2. Our study provides comprehensive m^5^C profiles of mammalian transcriptomes and suggests an essential role for m^5^C modification in mRNA export and post-transcriptional regulation.

## Introduction

The identification of fat mass and obesity-associated (FTO) as the first discovered RNA m^6^A demethylase^1^ established the reversible nature of m^6^A modification. Since then, accumulating lines of evidence have pointed to a broad effect of m^6^A on mRNA metabolism^2,3,4,5,6,7,8,9,10,11,12,13,14^. More recently, another two RNA modifications, pseudouridine (ψ)^15,16,17^ and *N*1-methyladenosine (m^1^A)^18,19^, have also been shown to play a vital role in posttranscriptional gene regulation. All these lines of emerging evidence point to the logical speculation that reversible RNA modifications may serve as novel epigenetic markers with profound biological significance in RNA metabolism.

Another important RNA modification, 5-methylcytosine (m^5^C), was first identified in stable and highly abundant tRNAs and rRNAs^20,21,22^. Subsequently, many known or novel m^5^C sites have been validated by using advanced high-throughput techniques combined with next-generation sequencing (NGS). These include RNA bisulfite sequencing (RNA-BisSeq)^22,23^, m^5^C-RNA immunoprecipitation (RIP)^24^, 5-azacytidine-mediated RNA immunoprecipitation (Aza-IP)^25^ and methylation-individual-nucleotide-resolution crosslinking and immunoprecipitation (miCLIP)^26^. Based on available published data, a clear view of m^5^C modification in non-coding RNAs (ncRNAs) has been established, and many m^5^C sites have also been identified in mRNAs^23,25,26,27,28^. However, the knowledge about the prevalence and transcriptome-wide distribution of m^5^C in different species and tissues is still very limited. One recent study observed that m^5^C sites preferentially occur in the vicinity of the translational start codon in mouse embryonic stem (ES) cells and brain^27^. However, this feature was not detected in *Arabidopsis*^28^. Therefore, it is crucial to clarify how m^5^C modifications are distributed on mRNAs in different cell types, tissues, and organisms, and to identify and characterize the roles of the protein factors responsible for adding/removing/reading these modifications in order to gain better understanding of the potential significance of m^5^C modification for RNA metabolism.

In this study, we mapped transcriptome-wide m^5^C profiles in human HeLa cells and multiple mouse tissues using RNA-BisSeq. The distributive analysis showed that m^5^C sites were mainly enriched in the CG context and also in regions immediately downstream of translation initiation sites of mRNAs, and displayed conserved, tissue-specific and dynamic features in mammalian transcriptomes. We further identified that NSUN2 is the main enzyme catalyzing m^5^C formation, while Aly/REF export factor (ALYREF, an mRNA transport adaptor, also named THOC4) functions as a specific mRNA m^5^C-binding protein regulating mRNA export. Our data provide a comprehensive description of m^5^C methylomes in mammalian transcriptomes and further illustrate an essential role of m^5^C in regulating mRNA export.

## Results

### Distribution profiles of m^5^C in mRNAs

**Figure 1:**
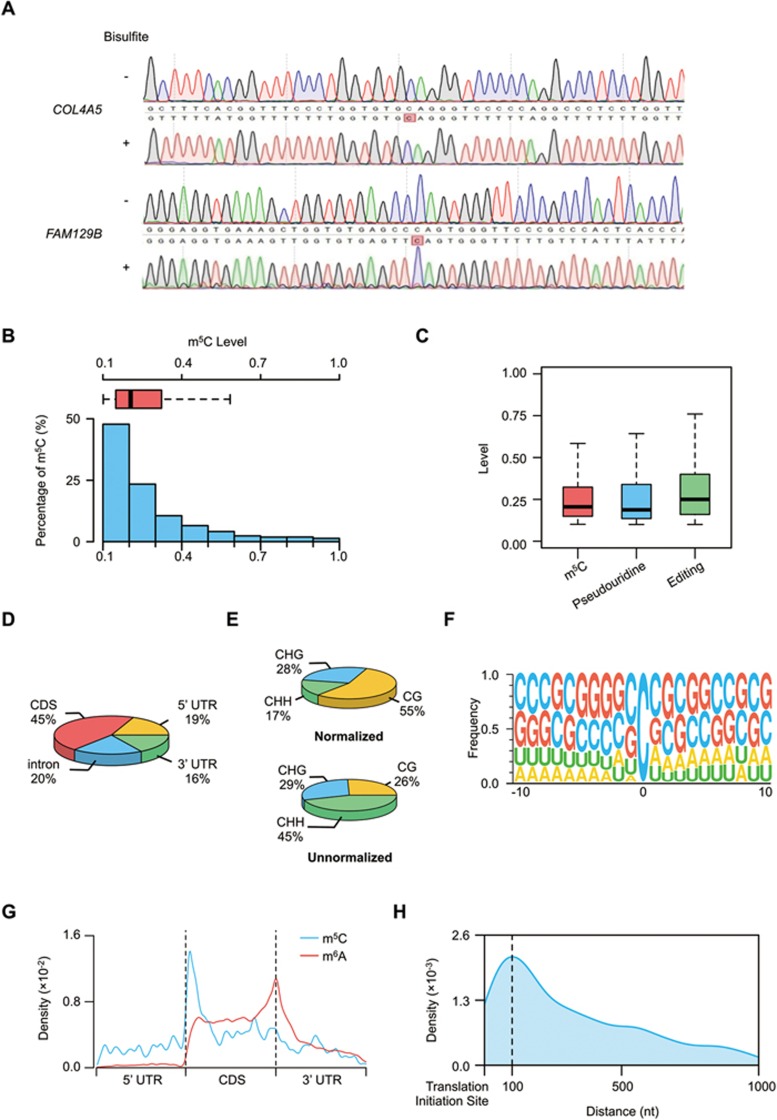
Distribution profiles of m^5^C in mRNAs. **(A)** Sanger-based validation of representative m^5^C sites. m^5^C sites within *COL4A5* (chrX:107911644) and *FAM129B* (chr9:130268749) (hg19) identified by RNA-BisSeq were validated. cDNA was amplified by PCR using normal primers for untreated mRNAs and specific primers for bisulfite-treated mRNAs. **(B)** Histogram and box plot showing the mRNA m^5^C levels. Methylation levels of majority of m^5^C sites were < 40%. **(C)** Box plot showing the levels of mRNA m^5^C, pseudouridine and editing. **(D)** Transcriptome-wide distribution of mRNA m^5^C sites. Pie chart presenting the fraction of m^5^C sites within distinct mRNA regions: CDS, intron, 5′ UTR and 3′ UTR. **(E)** The normalized and unnormalized proportions of mRNA m^5^C sites identified in each sequence context: CG, CHG and CHH, where H = A, C, or U. The normalization was done by normalizing m^5^C numbers in each of three contexts to their individual context proportion within transcriptome. **(F)** Sequence frequency logo for the sequences proximal to mRNA m^5^C sites. **(G)** Distribution of m^5^C sites and m^6^A peaks along mRNA transcripts. The moving averages of percentages of mRNA m^5^C sites and m^6^A peaks were shown. **(H)** Distribution of m^5^C sites across CDS regions of mRNA transcripts.

To obtain a transcriptome-wide landscape of m^5^C profiles, we performed RNA-BisSeq analysis on RNA samples purified from human HeLa cells. Based on a recently described method^29^, we used ACT random hexamers devoid of Gs to prime the reverse transcription (RT) of bisulfite-treated poly(A)-enriched RNA samples aiming to avoid copying inefficiently deaminated RNA templates. Since RNA-BisSeq cannot distinguish m^5^C from its potential oxidation product of hm^5^C, we applied UHPLC-MRM-MS/MS (ultra-high-performance liquid chromatography-triple quadrupole mass spectrometry coupled with multiple-reaction monitoring) to verify the presence of m^5^C and hm^5^C in mammalian mRNAs. Unlike the readily detectable m^5^C, we observed hm^5^C levels to be very low in human and mouse mRNAs (Supplementary information, Figure S1A), consistent with a recent report^30^. To ensure efficient conversion of RNA-BisSeq samples, the threshold for global conversion rate (C to T) was set to > 99.6% using dihydrofolate reductase (*Dhfr*) as the methylation conversion control (Supplementary information, Table S1). Validation of mRNA m^5^C sites identified by RNA-BisSeq was undertaken by randomly selecting several m^5^C sites for subsequent PCR amplification and Sanger sequencing. All of the Cs were demonstrated to be converted to Ts except the methylated sites detected by RNA-BisSeq (Figure 1A and Supplementary information, Figure S1B). Thus, by choosing the ACT random hexamers and the most stringent conversion conditions, the RNA-BisSeq approach was capable of faithfully identifying m^5^C sites in transcriptomes.

The data from RNA-BisSeq were shown to be highly reproducible between independent replicates (Supplementary information, Figure S1C, S1D, Tables S1 and S2). A total of 5 399 m^5^C sites within 2 243 RNA molecules identiﬁed in both HeLa replicates (“high-conﬁdence” set) were used in subsequent bioinformatic analyses. Among the m^5^C sites identified, a majority (94%, 5 063/5 399) were found to occur within 1 995 mRNAs (Supplementary information, Figure S1E). The remaining 336 m^5^C sites were mapped to diverse types of ncRNAs, predominantly to lncRNAs, including pseudogene transcripts, processed transcripts, lincRNAs, natural antisense transcripts and others (Supplementary information, Figure S1F). Notably, the median methylation level of mRNA m^5^C sites is about 20.5% (Figure 1B), similar to the degree of mRNA pseudouridylation or editing (Figure 1C), two other types of RNA modifications that have been quantified throughout the transcriptome at single-nucleotide resolution^16,31^. The distribution profile of m^5^C sites in mRNA was then examined and the most abundant m^5^C modification was found to be in coding sequence (CDS, 45%) (Figure 1D). Interestingly, mRNA m^5^C sites were 55% in CG regions, 28% in CHG regions and 17% in CHH regions (where H = A, C, U) after normalizing m^5^C numbers in each of three contexts to their individual context proportion within transcriptome (Figure 1E and Supplementary information, Figure S1G). A sequence frequency logo demonstrates that m^5^C sites, similar to m^1^A^18^, are embedded in CG-rich environments (Figure 1F). We next determined the enrichment feature of m^5^C sites in mRNA transcripts. Intriguingly, in addition to distribution in the vicinity of the binding regions for Argonaute proteins within 3′ UTRs^23^, we further observed that m^5^C sites are also enriched in regions immediately downstream of translation initiation sites (Figure 1G). This is similar to a recently published observation in mouse ES cells^27^ and different from the distribution of m^6^A that is enriched near stop codons and in 3′ UTRs^2,3^. A peak of the numbers of m^5^C sites was observed around 100 nucleotides after translation initiation sites across the whole length of CDS regions (Figure 1H), whereas no obvious “C” enrichment near translation initiation sites along mRNAs was observed (Supplementary information, Figure S1H). Moreover, there is no obvious correlation between m^5^C levels and mRNA abundance (Supplementary information, Figure S1I), suggesting that m^5^C probably participates in mRNA posttranscriptional processing. To gain insight into the potential function of m^5^C, we performed gene ontology (GO) enrichment analysis on m^5^C-containg mRNAs and found that they are involved in a variety of biological pathways (Supplementary information, Figure S1J), suggesting a potential role of mRNA m^5^C modification in regulating diverse functions of the organism.

### Tissue-specific pattern of mRNA m^5^C methylome

**Figure 2:**
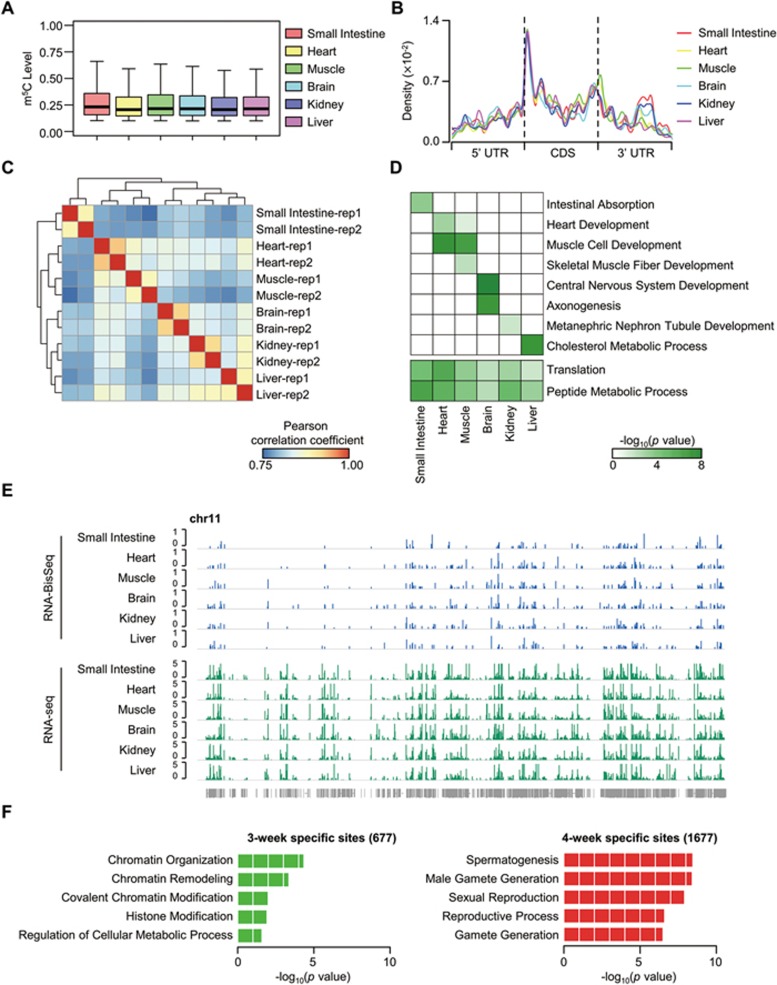
Tissue-specific mRNA m^5^C methylomes and dynamic features of mRNA m^5^C during testis development. **(A)** Boxplots showing the distributions of mRNA m^5^C levels across mouse tissues. **(B)** Distribution of m^5^C sites along mRNA transcripts in each tissue. The moving averages of mRNA m^5^C site percentage were shown. **(C)** Hierarchical clustering of Pearson correlation coefficient across mouse tissues, calculated by pairwise comparison of m^5^C levels. **(D)** Heatmap representing the combination of representative GO term enrichment (top: tissue-specific functions; bottom: common functions) in m^5^C-containing mRNAs in each tissue. Green to white color: high to low levels of GO term enrichment. **(E)** Browser representation of m^5^C levels and mRNA abundance within chromosome 11 across mouse tissues. **(F)** Gene ontology analysis of mRNAs with specific m^5^C sites in 3- or 4-week stage testis.

To further define the features of m^5^C distribution in mammalian mRNA methylomes, we performed deep RNA-BisSeq and RNA-seq in six mouse tissue samples, including small intestine, heart, muscle, brain, kidney and liver. The UHPLC-MRM-MS/MS approach was used to prove the presence of m^5^C in mouse tissue mRNAs (Supplementary information, Figure S2A). A range of 2 540-4 371 m^5^C sites within 1 016-1 655 mRNAs were identified in each of six mouse tissues (Supplementary information, Figure S2B, Tables S1 and S3). In total 9 788 sites were detected within 3 904 mRNAs, and among these, 755 sites within 223 mRNAs were commonly present in all six tissues. The median methylation level of mRNA m^5^C in each mouse tissue was between 20.6% and 23.2% (Figure 2A), similar to the level in human HeLa cells (Figure 1B). Additionally, these m^5^C sites were mainly present in CG-rich regions and in CDS regions (Supplementary information, Figure S2C-S2E), and moreover, they were enriched in regions immediately downstream of translation initiation sites (Figure 2B and Supplementary information, Figure S2F). This accords with the distribution profile feature of mRNA m^5^C in HeLa cells. Therefore, the distribution profile of m^5^C in mRNA is well-conserved in mammalian cells. To explore the tissue-specific features of mRNA m^5^C methylomes, we performed hierarchical clustering on m^5^C methylation levels. This revealed that tissues of similar origins (for example, heart and skeletal muscle) clustered together (Figure 2C). Functional enrichment analysis further revealed that m^5^C-containing mRNAs in each tissue participate in both common biological processes and tissue-specific functions (Figure 2D). Indication of tissue-specific patterns was given by the finding that m^5^C sites were not uniformly present in all six mouse tissues (Figure 2E). Accordingly, between 735 and 1 918 tissue-specific m^5^C sites were identified in each tissue and functional enrichment analysis revealed that the mRNAs modified in this way are closely associated with tissue-specific functions (Supplementary information, Figure S2G). Considering that mRNA abundance may influence the identification of m^5^C sites, we then selected mRNAs expressed in all six tissues and identified between 347 and 628 m^5^C sites specific for each of six tissues (Supplementary information, Figure S2H).

### Dynamic m^5^C modifications during testis development

To determine whether m^5^C methylation occurs dynamically during tissue development, we subjected testis mRNA samples from 3- and 4-week-old mice to deep RNA-BisSeq and RNA-seq (Supplementary information, Tables S1 and S3). We identified 3 348 and 4 366 m^5^C sites in 1 265 and 1 791 mRNAs from the respective 3- and 4-week stages. Within mRNAs expressed in both stages, 677 and 1 677 m^5^C sites were specifically present in 3- and 4-week samples, respectively, suggesting that m^5^C methylation is highly dynamic during testis development. Functional annotation of mRNAs with specific m^5^C sites in each stage indicated that they correlated with stage-specific functions. For example, mRNAs with specific m^5^C sites in the 4-week stage were significantly associated with spermatogenesis (Figure 2F), consistent with the undergoing spermatogenesis at this stage^32^. Quantitative analysis of common m^5^C sites between the two stages revealed that stage-specific regulation of m^5^C level is found in 37% of m^5^C commonly modified mRNAs. Functional annotation indicated that these mRNAs were involved in stage-specific functions (Supplementary information, Figure S2I and S2J). Collectively, these results suggest dynamic regulation of m^5^C modification during tissue development.

### NSUN2 as the main mRNA m^5^C methyltransferase

NSUN2 has been shown to catalyze m^5^C formation in RNAs^23,25,26,33,34^ and functionally linked to cell cycle control, (stem) cell differentiation and proliferation, and development^35^. To uncover the potential methyltransferase(s) responsible for mRNA m^5^C formation in whole transcriptome, several candidates of the NOP/SUN (NSUN) family members including *NSUN1*, *NSUN2*, *NSUN5* and *NSUN6*, were either suppressed or overexpressed in HeLa cells. The abundance of m^5^C in mRNAs was then measured by UHPLC-MRM-MS/MS. Interestingly, even though all NSUN family members contain conserved catalytic sites and releasing sites (Supplementary information, Figure S3A), only NSUN2 significantly affected the m^5^C levels in mRNAs rather than in total RNAs (Supplementary information, Figure S3B-S3G). An NSUN2 mutant with mutated releasing (cysteine 271) and catalytic (cysteine 321) sites^26,36^ totally lost its regulatory effect on mRNA m^5^C levels (Supplementary information, Figure S3H-S3J). Transcriptome-wide m^5^C analyses were then performed on control and NSUN2-knockdown HeLa cells by RNA-BisSeq and RNA-seq (Supplementary information, Figure S3K and Tables S1 and S2). The findings showed that 2 016 m^5^C sites present in control HeLa cells had reduced methylation levels in NSUN2-knockdown cells (Supplementary information, Figure S3L). Consistent with the finding of Squires *et al*.^23^, NSUN2 serves as the main mRNA m^5^C methyltransferase.

### Specifically binding of ALYREF to mRNA m^5^C sites

**Figure 3:**
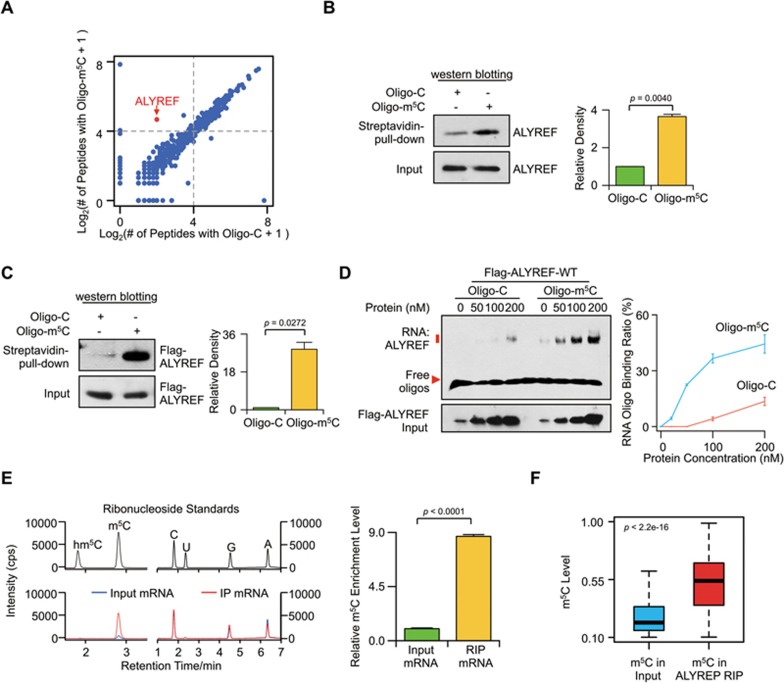
ALYREF is a specifi c mRNA m^5^C-binding protein. **(A)** Scatter plot of proteins bound to Oligo-m^5^C versus Oligo-C RNA oligos. The plot was based on the average peptide numbers of proteins detected in both replicates. Enriched ALYREF protein was highlighted. **(B)** Demonstration of endogenous ALYREF pulled down by biotin-labeled RNA oligonucleotides containing m^5^C (Oligo-m^5^C). Left, western blotting; right, quantification level. **(C)** Demonstration of purified Flag-ALYREF pulled down by biotin-labeled Oligo-m^5^C. Left, western blotting; right, quantification level. **(D)** EMSA (left) and line graph quantification (right) showing the binding ability of purified Flag-ALYREF-WT with Oligo-m^5^C or Oligo-C. 100 nM of RNA Oligo-m^5^C or Oligo-C was incubated with different concentrations of Flag-ALYREF-WT protein. The RNA binding ratio was calculated by (RNA-protein)/((free RNA) + (RNA-protein)). Error bars indicate SEM (*n* = 3). *P* values were calculated using Student's *t*-test. **(E)** UHPLC-MRM-MS/MS chromatograms (left) and quantification (right) of m^5^C in input and *in vitro* ALYREF-RIP mRNA samples. **(F)** Boxplot showing m^5^C level of methylation sites detected in both input and *in vivo* ALYREF-RIP mRNA samples. *P* values were calculated using Mann-Whitney U test.

To search for specific mRNA m^5^C-binding proteins, we performed RNA affinity chromatography and mass spectrometry analyses using biotin-labeled oligonucleotides with or without m^5^C. ALYREF/THOC4, the mammalian mRNA export adaptor, was observed to be enriched in both replicate complexes immunoprecipitated by m^5^C-containing oligonucleotide (peptideatlas access number PASS00817) (Figure 3A, 3B and Supplementary information, Figure S4A and S4B). Consistently, both *in vitro* pull-down and electrophoretic mobility shift assays (EMSA) illustrated that ALYREF has a significantly higher level of binding ability to m^5^C-modified oligonucleotide than to unmethylated control (Figure 3C, 3D and Supplementary information, Figure S4C). We also performed UHPLC-MRM-MS/MS and RNA-BisSeq analyses to define m^5^C enrichment in mRNAs immunoprecipitated by ALYREF. Relative to input mRNA control, *in vitro* ALYREF-RIP mRNAs showed approximately 8.7-fold higher levels of m^5^C modification (Figure 3E and Supplementary information, Figure S4D). Furthermore, RNA-BisSeq demonstrated that the m^5^C methylome of *in vivo* ALYREF-RIP mRNAs displayed an m^5^C enrichment in CG-rich regions and in regions immediately downstream of translation initiation sites (Supplementary information, Figure S4E, S4F, Tables S1 and S4) with levels of m^5^C that were significantly higher than those of the input mRNA (Figure 3F).

**Figure 4:**
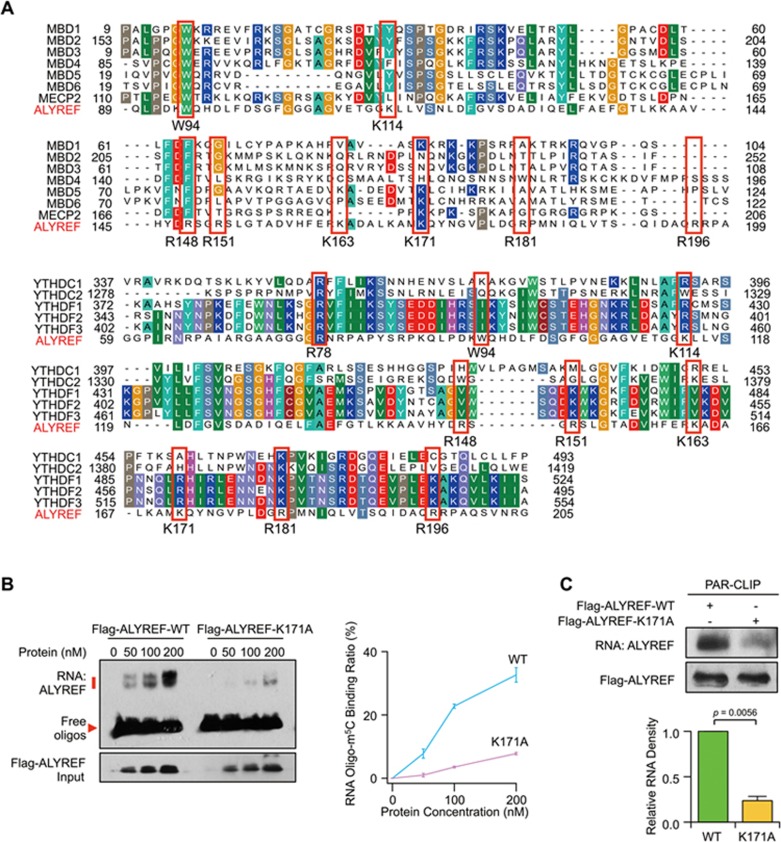
ALYREF specifically binds to mRNA m^5^C sites via K171. **(A)** Multiple sequence alignments of ALYREF (GenBank: NP_005773.3), MBD family members MBD1-6 (GenBank: NP_001191065.1, NP_003918.1, NP_001268382.1, NP_001263199.1, NP_060798.2, and NP_443129.3) and MeCP2 (GenBank: NP_001104262.1) (top). Multiple sequence alignments of ALYREF and YTH family members: YTHDC1, YTHDC2, YTHDF1, YTHDF2 and YTHDF3 (GenBank: NP_001026902.1, NP_073739.3, NP_060268.2, NP_001166299.1, and NP_001264742.1) (bottom). The relatively conserved amino acids used for constructing mutants are highlighted in red boxes. **(B)** EMSA (left) and line graph quantification (right) showing the RNA-binding ability of Flag-ALYREF wild-type (WT) or mutant (K171A) to Oligo-m^5^C. **(C)** PAR-CLIP assay (top) and quantification (bottom) of RNAs pulled down by Flag-ALYREF-WT or -K171A in HeLa cells. *P* values were calculated by Student's *t*-test. Data shown are mean ± SEM (*n* = 3).

To identify the essential amino acids responsible for the specific binding of ALYREF to m^5^C sites, we aligned the ALYREF protein sequence with MBD and YTH family members that can recognize 5-methylcytosine (5mC) in DNA^37^ and m^6^A in RNA^2,4,10,14,38^, respectively. Several relatively conserved amino acid residues (Figure 4A) were selected, and then individually mutated in purified Flag-tagged recombinant ALYREF protein for EMSA (Supplementary information, Figure S4C and S4G). This revealed that the K171 mutation led to a dramatically reduced level of ALYREF binding to m^5^C-containing oligonucleotide (Figure 4B and Supplementary information, Figure S4C). Consistently, PAR-CLIP revealed that the *in vivo* RNA-binding ability of ALYREF was diminished by the K171 mutation (Figure 4C and Supplementary information, Figure S4H). This indicates that ALYREF is a specific binding protein of m^5^C methylated mRNA.

### ALYREF nuclear-cytoplasmic shuttling regulated by NSUN2

**Figure 5:**
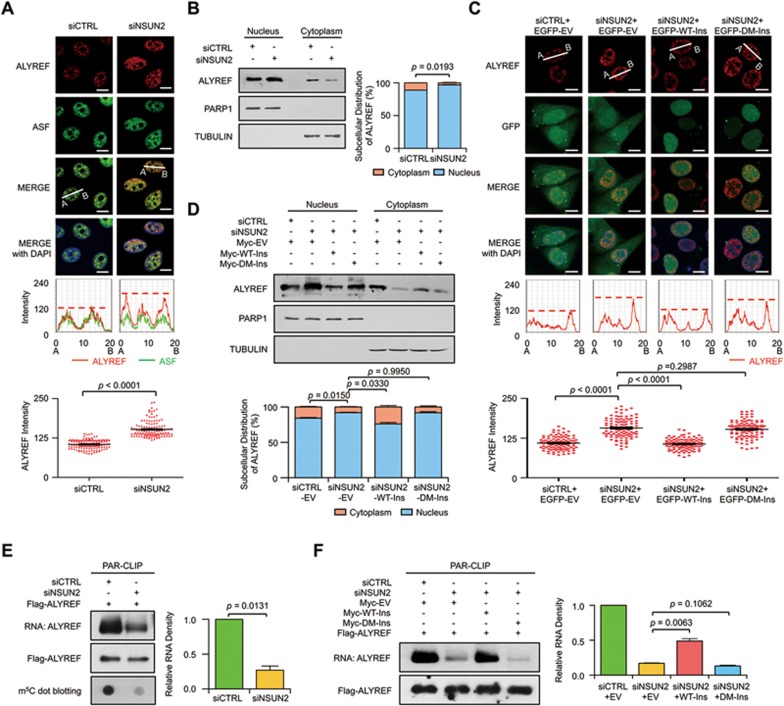
NSUN2 regulates nuclear-cytoplasmic shuttling and RNA-binding ability of ALYREF. **(A)** Immunofluorescence staining of ALYREF (red color) and ASF (green color) upon NSUN2 knockdown (top); line scan graphs (middle) and peak density quantification of line scan graphs (bottom) for ALYREF are also shown. Scale bar, 10 μm. Error bars indicate SEM (*n* = 120). **(B)** Western blotting (left) and quantification (right) of nuclear and cytoplasmic distribution of ALYREF in control and NSUN2-knockdown HeLa cells. The protein loading for the cytoplasmic fraction is about 2.5-fold higher than that for the nuclear fraction. PARP1 and TUBULIN serve as nuclear and cytoplasmic markers, respectively. Error bars indicate SEM (*n* = 3). **(C)** Immunofluorescence staining of ALYREF (red color) in NSUN2-knockdown HeLa cells transfected with control EGFP (EGFP-EV), EGFP-tagged siNSUN2-insensitive wild-type NSUN2 (EGFP-WT-Ins) or mutant (EGFP-DM-Ins) plasmids (top); line scan graphs (middle) and peak density quantification of line scan graphs (bottom) for ALYREF are also shown. Scale bar, 10 μm. Error bars indicate SEM (*n* = 120). **(D)** Western blotting (top) and quantification (bottom) of nuclear and cytoplasmic distribution of ALYREF in NSUN2-knockdown HeLa cells transfected with empty Myc expression vector (Myc-EV), Myc-tagged siNSUN2-insensitive wild-type NSUN2 (Myc-WT-Ins), or Mutant (Myc-DM-Ins). The protein loading for the cytoplasmic fraction is about 2.5-fold higher than that for the nuclear fraction. PARP1 and TUBULIN serve as nuclear and cytoplasmic markers, respectively. Error bars indicate SEM (*n* = 3). **(E)** PAR-CLIP assay (left) and quantification (right) of RNA pulled down by Flag-ALYREF upon NSUN2 knockdown. RNA labeled with biotin at 3′ end of RNA (End Biotinylation Kit, Thermo) was visualized by the chemiluminescent nucleic acid detection module. m^5^C-modified RNAs were visualized by dot blotting using m^5^C antibody. Error bars indicate SEM (*n* = 3). **(F)** Rescue PAR-CLIP assay (left) and quantification (right) of RNA pulled down by Flag-ALYREF in NSUN2-knockdown HeLa cells transfected with Myc-EV, Myc-WT-Ins, or Myc-DM-Ins. RNA labeled with biotin at 3′ end of RNA (End Biotinylation Kit, Thermo) was visualized by the Chemiluminescent nucleic acid detection module. Error bars indicate SEM (*n* = 3). *P* values were calculated by Student's *t*-test.

ALYREF has been reported to undergo nuclear-cytoplasmic shuttling and reside in nuclear speckles enriched with pre-mRNA processing factors^39,40^. The findings that ALYREF directly binds to m^5^C-modified mRNAs led us to examine whether nuclear-cytoplasmic shuttling of ALYREF is regulated by NSUN2. A dramatically increased level of ALYREF nuclear speckle staining was observed in NSUN2-knockdown cells, while the total protein abundance was unchanged (Figure 5A and Supplementary information, Figure S5A-S5D). In support, the enhanced nuclear retention and subsequently decreased cytoplasmic localization of ALYREF upon NSUN2 knockdown were also observed (Figure 5B). Moreover, these dysregulated subcellular distributions of ALYREF upon NSUN2 knockdown could be rescued by reconstitution with wild-type but not m^5^C methyltransferase-defective NSUN2 (Figure 5C and 5D and Supplementary information, Figure S5E and S5F). This provides direct evidence that the catalytic activity of NSUN2 is required for the regulation of ALYREF nuclear-cytoplasmic shuttling.

### m^5^C-dependent ALYREF RNA binding

We then tested whether the RNA-binding affinity of ALYREF is regulated by NSUN2 by PAR-CLIP and *in vitro* RNA end biotin-labeling assays. These experiments revealed that even though ALYREF nuclear retention was significantly enhanced upon NSUN2 silencing, its RNA-binding affinity was markedly decreased (Figure 5E and Supplementary information, Figure S5G) and this could be restored by wild-type but not m^5^C methyltransferase-defective NSUN2 (Figure 5F and Supplementary information, Figure S5H). In contrast, the RNA-binding affinity of NSUN2 was not affected by ALYREF silencing (Supplementary information, Figure S5I and S5J). These findings clearly indicated that the RNA-binding affinity of ALYREF is m^5^C-dependent.

### mRNA export promoted by m^5^C modification

**Figure 6:**
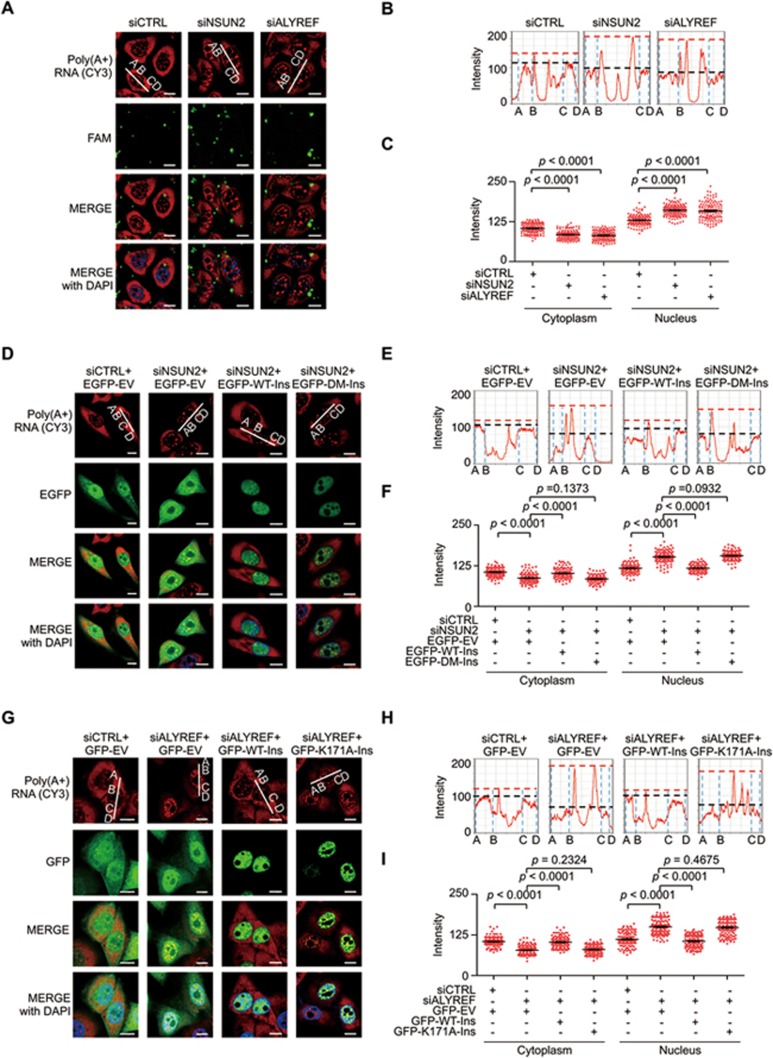
Involvement of m^5^C in mRNA export regulation. **(A**-**C)** Fluorescence *in situ* hybridization (FISH) analysis of mRNAs (red) in the control, NSUN2- or ALYREF-deﬁcient HeLa cells **(A)**; line scan graphs **(B)** and peak density quantification of line scan graphs **(C)** for mRNAs are shown. Green: FAM-labeled siRNAs. The red and black dash lines **(B)** represent the peak densities of nuclear and cytoplasmic mRNAs, respectively. Scale bar, 10 μm. Error bars indicate SEM (*n* = 120). **(D**-**I)** FISH analysis of mRNAs (red color) in NSUN2 **(D**-**F)** or ALYREF **(G**-**I)** knockdown HeLa cells reconstituted with control vector, EGFP/GFP-tagged wild-type or mutant forms of NSUN2 **(D**-**F)** or ALYREF **(G**-**I)**; line scan graphs **(E**, **H)** and peak density quantification of line scan graphs **(F**, **I)** for mRNAs are shown. The red and black dash lines **(E**, **H)** represent the peak densities of nuclear and cytoplasmic mRNAs, respectively. Scale bar, 10 μm. Error bars indicate SEM (*n* = 120). *P* values were calculated by Student's *t*-test.

Previous evidence of ALYREF as an mRNA export adaptor^39^, together with our findings that ALYREF specifically binds to m^5^C sites in mRNA, supports the hypothesis that m^5^C modification probably contributes to the regulation of mRNA export. To test this hypothesis we examined mRNA export following modulation of NSUN2 or ALYREF expression using fluorescence *in situ* hybridization (FISH). As expected, NSUN2 knockdown significantly increased nuclear mRNA content (Figure 6A-6C and Supplementary information, Figure S6A and S6B) that could be recovered to control levels by reconstitution of wild-type but not m^5^C methyltransferase-defective NSUN2 (Figure 6D-6F and Supplementary information, Figure S6C). Similarly, a higher level of nuclear mRNA staining could also be seen following ALYREF knockdown, an effect that could be suppressed by overexpression of wild-type but not m^5^C-binding defective ALYREF (Figure 6A-6C, 6G-6I and Supplementary information, Figure S6A and S6D).

**Figure 7:**
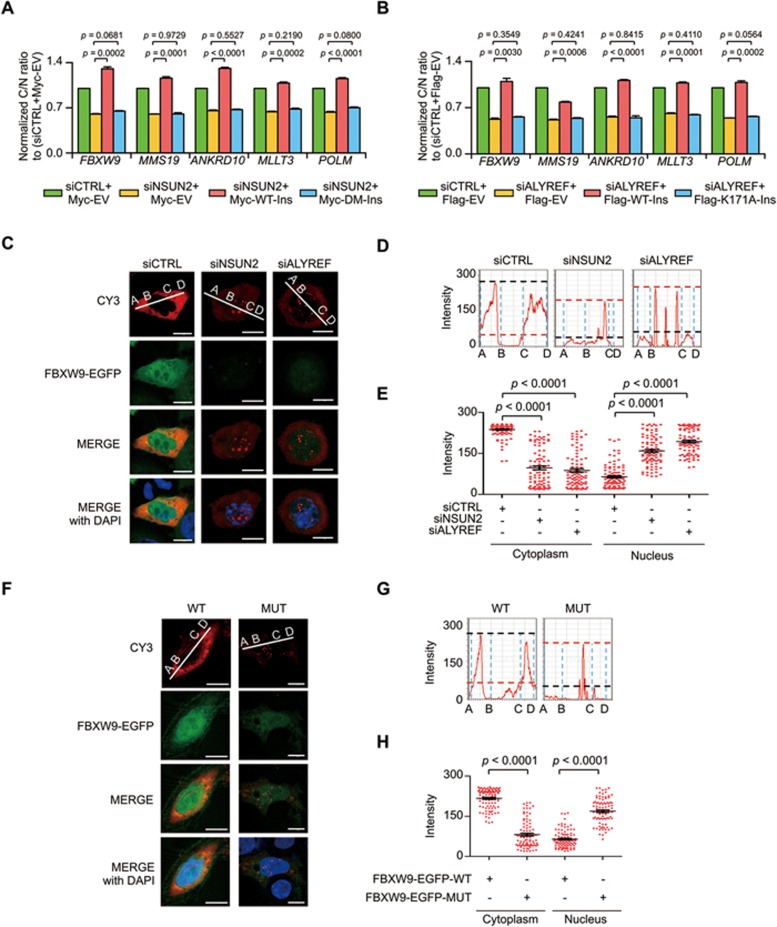
m^5^C recognition by ALYREF promotes mRNA export. **(A**, **B)** qPCR analysis of the relative cytoplasmic to nucleic ratios of NSUN2 target genes with m^5^C modification in NSUN2 **(A)** or ALYFEF **(B)** knockdown HeLa cells reconstituted with control vector, wild-type or mutant forms of NSUN2 **(A)** or ALYREF **(B)**. Error bars indicate SEM (*n* = 3). **(C**-**E)** FISH analysis of EGFP-tagged *FBXW9* Exon 1 minigene mRNAs (red color) in NSUN2 or ALYREF knockdown HeLa cells transfected with m^5^C site-containing wild-type FBXW9-EGFP minigene construct (FBXW9-EGFP-WT, **C**); line scan graphs **(D)** and peak density quantification of line scan graphs **(E)** for minigene mRNAs are shown. The minigene mRNA export was measured by Cy3-labeled oligonucleotide probes complementary to *EGFP* mRNAs. Scale bar, 10 μm. Error bars indicate SEM (*n* = 110). **(F)** Nuclear export of *FBXW9* minigene mRNAs in HeLa cells transfected with m^5^C site-containing (FBXW9-EGFP-WT) or mutant (FBXW9-EGFP-MUT) minigene plasmids was analyzed by FISH assay using Cy3-labeled oligonucleotide probes complementary to *EGFP* mRNAs. **(G**-**H)** Line scan graphs **(G)** and peak density quantification of line scan graphs for minigene mRNAs **(H)** are shown. Scale bar, 10 μm. Error bars indicate SEM (*n* = 110). *P* values were calculated by Student's *t*-test.

**Figure 8:**
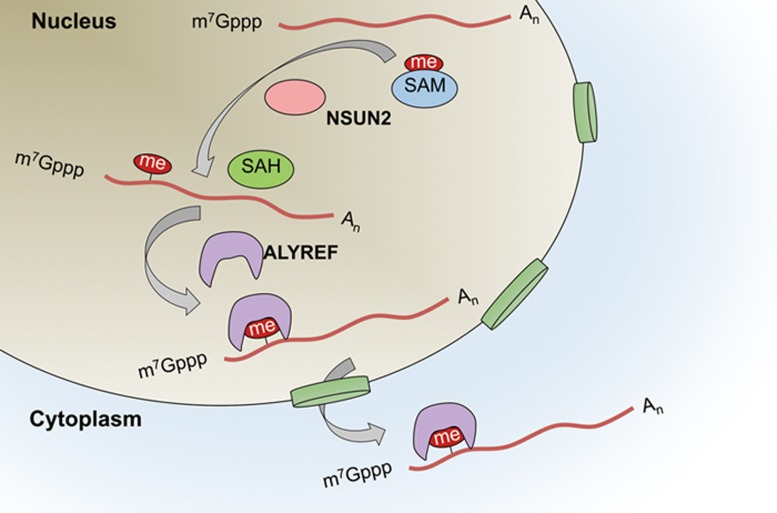
Working model showing dynamic regulation of m^5^C in mRNA. m^5^C formation is catalyzed by NSUN2. This modification provides a recognition target for ALYREF to mediate mRNA export from the nucleus.

To further validate this finding, the global effects of m^5^C-methylation upon mRNA export were examined in several randomly selected NSUN2 mRNA targets. This revealed that knockdown of either NSUN2 or ALYREF significantly decreased the cytoplasmic to nuclear ratios of mRNAs tested (Supplementary information, Figure S7A-S7C). This dysregulated nuclear export could be rescued only by reconstitution of wild-type NSUN2 or ALYREF but not by their individual mutants (Figure 7A, 7B and Supplementary information, Figure S7D-S7G). Interestingly, m^5^C-modified mRNAs not targeted by NSUN2 showed substantially suppressed nuclear export upon ALYREF but not NSUN2 depletion (Supplementary information, Figure S7A, S7B and S7H). Moreover, mRNAs without m^5^C modifications failed to show any obvious changes in nuclear export after knockdown of either NSUN2 or ALYREF (Supplementary information, Figure S7A, S7B and S7I). These findings illustrate that ALYREF is the main nuclear m^5^C reader that functions in promoting mRNA export. We further employed a minigene reporter assay in which the m^5^C-containing *FBXW9* Exon 1 minigene was fused to an EGFP tag to validate the above findings (Supplementary information, Figure S7J and S7K). mRNA export was measured by Cy3-labeled oligonucleotide probes complementary to *EGFP* mRNAs. Consistently, NSUN2 or ALYREF knockdown led to a significant decrease in nuclear EGFP protein expression versus a dramatic enhancement in nuclear mRNA-Cy3 signal intensity (Figure 7C-7E) that were also observed when a m^5^C site in *FBXW9* Exon 1 minigene was mutated (Figure 7F-7H and Supplementary information, Figure S7L). However, *EGFP* mRNA abundance in HeLa cells was not affected by transfection with minigene plasmids containing m^5^C site or its mutant (Supplementary information, Figure S7M). Together, these results demonstrate that m^5^C modification is essential for mediating mRNA export (Figure 8).

## Discussion

In this study, we utilized an RNA-BisSeq approach to characterize the m^5^C methylomes in human and mouse transcriptomes. This has revealed the main features of m^5^C modifications: their prevalence and unique distribution along transcripts and their tissue-specific and dynamic nature in mRNA. We further demonstrated that m^5^C is specifically recognized by the mRNA export adaptor ALYREF in experiments *in vitro* and *in vivo*. Finally, m^5^C was proved to promote mRNA export coordinately regulated by its methyltransferase NSUN2 and binding partner ALYREF. Thus, m^5^C and its associated proteins participate in the dynamic regulation of selective mRNA export in mammalian cells.

m^5^C modification in ncRNAs has been well-documented. However, its distribution profile in mRNAs remains unclear even though many m^5^C sites have been defined in mRNAs^23,25,26,27,28^. In this study, m^5^C sites were observed to be enriched near the translation initiation sites of mRNAs, and these patterns of its distribution are highly conserved in human and mouse cells and tissues. A similar pattern of distribution of m^5^C was also reported recently in mouse ES cells and brain tissues^27^, but not detected in *Arabidopsis*^28^. This suggests that the m^5^C modification, similar to the m^6^A modification, has a distinct distribution pattern in plant mRNAs from that of mammals^41^. This species-specific difference was also observed in an association analysis between mRNA abundance and m^5^C level where a small negative correlation was found in *Arabidopsis*^28^, but not in mammals^26,34^.

mRNA modifications apparently exert their functions through recruiting their specific binding proteins, as evidenced by the findings that m^6^A-modified RNA can be selectively bound by different YTH family members, leading to the regulation of mRNA splicing, translation and degradation^4,10,14^. However, reader proteins that specifically recognize the m^5^C modification remain to be characterized even though their identification is critical for a better understanding of the biological significance of m^5^C. In this study, we have demonstrated that ALYREF, an mRNA export adaptor, serves as a specific m^5^C-binding protein (Figures 3 and 4) and functions in promoting mRNA export (Figure 8).

Two main mechanisms of eukaryotic mRNA export have been well-characterized. The first is the NXF1-dependent bulk mRNA export pathway in which an NXF1-NXT1 heterodimer binds to mRNA via the TREX-1/2 complex. TREX-1 consists of ALYREF/THOC4, UAP56, CIP29, PDIP3, ZC11A, UIF, and the THO subcomplex (THOC1/2/3/5/6/7), in which ALYREF functions as an adaptor^42,43,44,45,46,47^. The TREX-2 complex contains GANP, ENY2, CETN2/CETN3, PCID2, and DSS1^48,49,50,51,52^. The second is the CRM1-dependent mRNA export pathway that utilizes three adaptor proteins including RNA-binding protein human antigen R (HuR)^53^, leucine-rich pentatricopeptide repeat protein (LRPPRC)^54^, and nuclear export factor 3 (NXF3)^55^. However, whether or not RNA modifications are involved in mRNA export remains unclear. In this study, we provided strong evidence that m^5^C is specifically recognized by ALYREF and consequently promotes selective mRNA export. Meanwhile, the dynamic nature of the m^5^C modification suggests that, similar to m^6^A^56^, the m^5^C sites in a substantial proportion of mRNAs can be dynamically modified under different physiological conditions, and the ALYREF-dependent pathway serves as one of the main mechanisms for the selective export of m^5^C-modified mRNAs. However, export of m^5^C-unmodified mRNAs may be mediated by ALYREF-independent pathways.

In summary, our findings illustrate that the m^5^C modification is well-conserved and dynamically regulated in cellular mRNAs. It may inﬂuence a wide variety of biological functions through regulating RNA metabolism, in particular mRNA export. Our study provides a valuable resource for deciphering the potential biological significance of m^5^C and opens up new functions for m^5^C modification in mRNA metabolism.

## Materials and Methods

### Plasmids

Human *NSUN2* and *NSUN5* genes were amplified by PCR using human HeLa cDNA and subcloned into pcDNA3-Flag, pEGFP-C1 or pCMV-myc vector (Addgene). The following wild-type plasmids were constructed: pcDNA3-Flag-NSUN2-WT, pEGFP-C1-NSUN2 and pCMV-myc-NSUN2. Flag-tagged human NSUN1 and NSUN6 plasmids were purchased from Vigene Biosciences, China. Myc-Flag-tagged (pCMV6-Entry-ALYREF-Myc-DDK) and GFP-tagged (pCMV6-AC-ALYREF-GFP) human ALYREF plasmids were purchased from Origene Technologies, USA.

The mutant and siRNA insensitive plasmids were generated by introducing point mutations into wild-type plasmids using QuikChange Lightning Site-Directed Mutagenesis Kit (Agilent Technologies).

To generate the mRNA export minigene construct, *FBXW9* mRNA Exon 1 containing one m^5^C modification site (cytosine 215 from start codon AUG) was amplified from human cDNA and inserted into the pEGFP-N1 vector (GenBank: U55762) named pEGFP-FBXW9-WT. The cytosine (C, methylated site) was mutated to adenine (A) using pEGFP-FBXW9-WT as template to generate pEGFP-FBXW9-MUT. All the primers used for cloning are listed in Supplementary information, Table S5.

All plasmids were validated by DNA sequencing, and prepared with the NucleoBond Xtra Midi Plasmid Purification Kit (Machery Nagel).

### Cell culture, transfections and antibodies

The human cervical carcinoma cell line HeLa, human embryonic kidney cell line 293T, mouse uterine cervix cancer cell line U14 and mouse collecting tubular epithelial cell line M-1 were obtained from Cell Resource Center, Chinese Academy of Medical Sciences and cultured in DMEM (Gibco) supplemented with 10% FBS (Shanghai ExCell Biology Inc.) and 0.5% penicillin/streptomycin (Sigma). Plasmids were transfected with Polyethylenimine (PEI, Polysciences) at a ratio of 1:3 (m/v) in serum- and antibiotic-free DMEM. After incubation for 15 min at room temperature, the mixture was added to serum- and antibiotic-free culture medium. 6 h after transfection, the medium was replaced with complete DMEM. The cell lines are not among commonly misidentified cell lines, and were routinely checked for mycoplasma contamination.

Rfect siRNA Transfection Reagent (BIO-TRAN) was used for transfection of siRNA duplexes. Co-transfection of siRNA duplexes with expression plasmids in the PAR-CLIP or single mRNA export rescue assay was performed by electroporation using the LONZA Kit (VCA-1001, Lonza, Germany) according to the manufacturer's instructions. All the siRNA duplexes used in this study were designed and synthesized by GenePharma, China, and are listed in Supplementary information, Table S5.

Antibodies were purchased from the following commercial sources: rabbit anti-NSUN2 (Proteintech, 20854-1-AP), rabbit anti-ALYREF (Abcam, ab202894), mouse anti-ASF (Invitrogen, 324600), mouse anti-PARP1 (BD Pharmigenn, 66401A), mouse anti-TUBULIN (Sigma, T5293), rabbit anti-Flag (Sigma, F7425), mouse anti-ACTIN (Santa Cruz, SC65638), mouse anti-GFP (ABclonal, AE012) and mouse anti-m^5^C antibody (Abcam, ab10805).

### *In vitro* transcription of *Dhfr* RNA

The mouse *Dhfr* gene encoding full length Dhfr was amplified by PCR and subcloned into the pcDNA3-HA vector (Addgene) which contains a T7 promoter sequence at its 5′ terminus. Purified pcDNA3-T7-HA-Dhfr plasmid was subjected to an *in vitro* transcription reaction with MEGAscript T7 RNA polymerase (Ambion) at 37 °C for 4 h in a 100 μl reaction mixture, according to the manufacturer's instructions. The primers used for cloning are:

forward: 5′-ATACTCGAGATGGTTCGACCATTGAACTGC-3′

reverse: 5′-ATAAGAATGCGGCCGCTTAGTCTTTCTTCTCGTAGACTTC-3′.

### RNA preparation

C57BJ/6 male mice were purchased from WeiTongLiHua experimental animal technical company (Beijing, China) and euthanized by cervical dislocation. Freshly dissected tissues were immediately frozen with liquid nitrogen, ground into powder, and immediately homogenized using TRIzol^®^ Reagent (Ambion) for total RNA isolation. Mice of 3- and 4-weeks old were chosen for postnatal testis development studies. All procedures have been approved by the Institutional Animal Care and Use Committee (IACUC) of the Beijing Institute of Genomics. Enrichment of mRNA from total RNA was performed using Dynabeads^®^ mRNA Purification Kit (Ambion). For rRNA depletion, the purified mRNA was further treated using Ribominus Transcriptome Isolation Kit (Human/Mouse) (Invitrogen).

### Bisulfite conversion of RNA

RNA fragmentation and bisulfite conversion was performed as previously described^29^ with some modifications. In brief, 1 μg of rRNA-depleted mRNAs along with 5 ng *Dhfr* RNA as methylation conversion control were fragmented into ∼200-nucleotide-long fragments by incubating for 50 s at 90 °C in 10× RNA Fragmentation Reagent. The fragmentation reaction was stopped by addition of stop solution (Ambion), followed by ethanol precipitation. The RNA pellet was resuspended in 100 μl bisulfite solution (pH 5.1), which is a 100:1 mixture of 40% sodium bisulfite (Sigma) and 600 μM hydroquinone (Sigma) and subjected to heat incubation at 75 °C for 4 h. The reaction mixture was desalted by twice passing it through Micro Bio-spin 6 chromatography columns (Bio-Rad) and then desulfonated by incubation with an equal volume of 1 M Tris (pH 9.0) at 75 °C for 1 h. After ethanol precipitation, the RNAs were resuspended in 10 μl of RNase-free water and used for library construction or validation.

### Sanger sequencing of PCR products

For the validation of methylated sites, a 300 ng aliquot of sodium bisulfite converted RNA was reverse transcribed into cDNA using ACT random hexamers and Superscript III Reverse Transcriptase Kit (Invitrogen) according to the manufacturer's instructions. cDNA was amplified by PCR using normal primers for untreated mRNAs and specific primers for bisulfite-treated mRNAs. To facilitate sequencing, the T7 promoter sequence was fused to the 5′ termini of forward primers, and the SP6 promoter sequence was fused to the 5′ termini of reverse primers. PCR products were separated on an agarose gel and extracted using PCR/Gel Purification Kit (Bioline) followed by Sanger-based sequencing. The primers of individual candidate gene are listed in Supplementary information, Table S5.

### Library construction and sequencing of bisulfite-converted RNAs

cDNA libraries were constructed using the KAPA Stranded mRNA-Seq Kit (KAPA) with some modifications. In brief, reverse transcription was carried out using ACT random hexamers and Superscript III Reverse Transcriptase (Invitrogen) according to the manufacturer's instructions. Sequencing was performed on an Illumina HiSeq2500 instrument with paired end 125-bp read length.

### UHPLC-MRM-MS/MS analysis of mononucleosides

RNAs were digested with 0.1 U Nuclease P1 (Sigma) and 1.0 U calf intestinal phosphatase (NEB) in 50 μl reaction volume at 37 °C overnight. The mixture was filtered by ultra-filtration tubes (MW cutoff: 3 kDa, Pall, Port Washington, NewYork), then analyzed to detect m^5^C, hm^5^C, rC, rU, rG, and rA. The UHPLC-MRM-MS/MS analysis was performed with an Agilent 1290 UHPLC system coupled with a 6495 triple quadrupole mass spectrometer (Agilent Technologies). A Zorbax Eclipse Plus C18 column (100 mm × 2.1 mm I.D., 1.8 μm particle size, Agilent Technologies) was used for UHPLC separation of mononucleosides. The mass spectrometer was operated in the positive ion mode. A multiple reaction monitoring (MRM) mode was adopted: m/z 258→126 for m^5^C, m/z 274→142 for hm^5^C, m/z 244→112 for rC, m/z 245→113 for U, m/z 284→152 for rG and m/z 268→136 for rA. The injection volume for each sample was 5 μl, and the amounts of m^5^C and rC was calibrated by standard curves. For ribonucleosides standards, 2 (for *in vitro* ALYREF-RIP mRNA samples) or 5 fmol (for cells and mouse tissues) of each of m^5^C and hm^5^C, 20 fmol of each of C, U, G, and A, and 400 fmol of U were used^57^. For mRNA of cell and mouse tissues, 40 ng was used to analyze m^5^C and hm^5^C, and 0.02 ng was used to analyze C, U, G, and A. For *in vitro* ALYREF-RIP mRNAs samples, 0.5 ng was used to analyze m^5^C and hm^5^C, and 0.01 ng was used to analyze C, U, G, and A. Nitrogen was used as a nebulizing and desolvation gas of MS detection. The nebulization gas was set at 40 psi, the flow-rate of desolvation gas was 9 L/min, and the source temperature was set at 300 °C. Capillary voltage was set at 3 500 V. High purity nitrogen (99.999%) was used as collision gas. Each sample was analyzed for at least three times. Ribonucleoside standards of m^5^C and hm^5^C were purchased from TCI, China, and GRANLEN, China, respectively.

### Isolation of cytoplasmic and nuclear fractions

The cytoplasmic and nuclear protein fractionation procedure was performed as described previously^58^ with some modifications. In brief, cells were trypsinized and washed once with cold PBS, and then incubated with 5 volumes of buffer A (10 mM HEPES pH 7.9, 1.5 mM MgCl_2_, 10 mM KCl, 0.5 mM DTT) supplemented with 1× Protease Inhibitor Cocktail (Sigma) for 10 min on ice. The cells were centrifuged at 2000 rpm for 10 min at 4 °C. The pellet was resuspended in 2 volumes of buffer A and slowly forced through the 1 ml syringe needle for 10 strokes to ensure complete cell lysis. The homogenate was centrifuged at 2000 rpm for 10 min at 4 °C and the supernatant was mixed with 0.11 volume of buffer B (0.3 M HEPES pH 7.9, 1.4 M KCl and 0.03 M MgCl_2_), and centrifuged at 10 000 g for 60 min at 4 °C. The supernatant from this step was designated as the cytoplasmic fraction. The pellet collected from the 2 000 rpm centrifugation was subjected to a second centrifugation at 25 000 g for 20 min at 4 °C to remove cytoplasmic residuals. The pellet was then resuspended in 2 volume of buffer C (20 mM HEPES pH 7.9, 25% (v/v) glycerol, 0.42 M NaCl, 1.5 mM MgCl_2_, 0.2 mM EDTA, 0.5 mM phenylmethylsulfonyl fluoride (PMSF) and 0.5 mM DTT), vigorously forced through the 1 ml syringe needle for 10 strokes for complete lysis of nuclei, and then centrifuged at 25 000 g for 30 min at 4 °C. The supernatant was designated as the nuclear fraction. The nuclear and cytoplasmic fractions were analyzed by western blotting using PARP1 and TUBULIN as nuclear and cytoplasmic markers, respectively.

### Recombinant protein purification from mammalian cells

293T cells were transfected with Flag-tagged plasmids and harvested at 36 h post transfection with lysis buffer (20 mM Tris-HCl pH 7.4, 500 mM NaCl, 1% NP-40, 1× Protease Inhibitor Cocktail) followed by sonication on ice using a Sonic Dismembrator (Fisher Scientific) (10-15 cycles with 10 s pulse-on and 20 s pulse-off, 10% amplitude). After centrifugation at 14 000 rpm for 15 min, the supernatant was filtered using a 0.2 μm syringe filter (Acrodisc Syringe filters) and the clear lysate was incubated with anti-Flag M2 Affinity Gel (Sigma) by gently rotating for 4 h at 4 °C. The beads were then washed twice with lysis buffer and twice with TBS buffer (20 mM Tris-HCl pH 7.4, 150 mM NaCl), and then subjected to incubation with 3× Flag peptides (Biotool) by gently rotating for 1 h at 4 °C to elute the bound proteins. Two rounds of elution were performed to maximize the recovery. The purified proteins were condensed using VIVASPIN 500 (Sartorius Stedim Biotech) and confirmed by SDS-PAGE followed by Coomassie brilliant blue staining or western blotting.

### RNA affinity chromatography, mass spectrometry and western blotting analysis

The biotin-labeled RNA oligonucleotides with (Oligo-m^5^C) or without m^5^C (Oligo-C): 5′-biotin-GAGGUAUGAA**X**UGUAAGTT-3′ (X = C or m^5^C), were synthesized by the Chemical Synthesis Center of the National Institute of Biological Sciences, Beijing. *In vivo* RNA pull-down assays were carried out using HeLa cell nuclear extracts as previously described^2,4^ with some modifications. Briefly, HeLa cell nuclear extracts were pre-cleared for 1 h at 4 °C by incubation with streptavidin-conjugated magnetic beads (NEB) in binding buffer (50 mM Tris-HCl pH 7.5, 250 mM NaCl, 0.4 mM EDTA, 0.1% NP-40, 1 mM DTT) supplemented with 0.4 U/μl RNasin (Promega). Biotin-labeled RNA oligonucleotides were incubated with pre-cleared nuclear extracts for 2 h at 4 °C under gentle rotation together with streptavidin-conjugated magnetic beads which were pre-cleared by incubation with 0.2 mg/ml tRNA (Sigma) and 0.2 mg/ml BSA (Amresco) for 1 h at 4 °C under gentle rotation. Beads were washed three times with wash buffer (50 mM Tris-HCl pH 7.5, 250 mM NaCl, 0.4 mM EDTA, 0.1% NP-40, 1 mM DTT, 0.4 U/μl RNasin (Promega)). Samples were separated on Novex^®^ 4%-20% TBE gel (Thermo) and stained with Coomassie brilliant blue. The protein-containing gel slices were applied to mass spectrometry analysis (BGI). Two independent biological replicates were performed. The mass spectrometry data files have been uploaded to http://www.peptideatlas.org with the access number: PASS00817. For western blotting analysis, samples were separated on SDS-PAGE and transferred onto PVDF membrane. After blocking with 5% non-fat dried milk in TBST for 1 h, the membrane was then incubated for 1 h at 4 °C with anti-ALYREF polyclonal antibody (Abcam, ab202894) diluted at 1:2 000 in 5% milk. Protein levels were visualized using ECL Western Blotting Detection Kit (GE Healthcare).

### Dot blotting

Synthesized biotin-labeled RNA oligonucleotides with or without m^5^C were quantified using UV spectrophotometry. Equal amounts of RNA oligos were loaded onto the positive charged nylon transfer membrane (GE Healthcare) fixed on the Bio-Dot Apparatus (Bio-Rad). For detection of m^5^C levels in Flag-ALYREF-RIP RNAs before and after NSUN2 knockdown, equal volumes of RNAs were loaded. After UV crosslinking for 3 min at 254 nm, the membrane was blocked with 5% non-fat dried milk in TBST followed by incubation with the primary mouse anti-m^5^C antibody (Abcam, ab10805) and HRP-conjugated Goat anti-mouse IgG (DakoCytomationn, p0161) secondary antibody. RNA levels were visualized by enhanced chemiluminescence (GE Healthcare). For biotin detection, the membrane was detected by chemiluminescent nucleic acid detection module (Thermo) following the manufacturer's instructions.

### *In vitro* RIP assay

*In vitro* RNA pull-down assay was carried out as previously described^2,4^ with some modifications. In brief, 10 pmol of purified Flag-ALYREF protein and 10 pmol of biotin-labeled RNA oligonucleotides with (Oligo-m^5^C) or without m^5^C (Oligo-C) were incubated with 15 μl streptavidin-conjugated magnetic beads (NEB) in binding buffer (50 mM Tris-HCl pH 7.5, 250 mM NaCl, 0.4 mM EDTA, 0.1% NP-40, 1 mM DTT) supplemented with 0.4 U/μl RNasin (Promega) for 1 h at 4 °C. After washing three times with binding buffer, the RNA-protein pull-down complexes were separated on the NuPAGE^®^ Novex^®^ 4%-20% TBE gel (Thermo), and immunoblotted with anti-Flag antibody (Sigma, F7425).

For fragmented mRNAs pull down assays, 20 μg purified mRNAs were fragmented into ∼100 nt length and then incubated with 20 μg Flag-ALYREF protein in binding buffer (50 mM Tris-HCl pH 7.5, 250 mM NaCl, 0.4 mM EDTA, 0.1% NP-40, 1 mM DTT, 0.4 U/μl RNasin (Promega)) for 1 h on ice. The protein-RNA complex was then incubated with anti-Flag M2 magnetic beads (Sigma) for 2 h at 4 °C with rotation. After washing three times with binding buffer, the protein-RNA-beads complex was digested with 4 μg/μl proteinase K (Roche) in 200 μl PK buffer (100 mM Tris-HCl pH 7.5, 50 mM NaCl, 10 mM EDTA) for 20 min at 37 °C following by incubation with 200 μl PK-urea buffer (100 mM Tris-HCl pH 7.5, 50 mM NaCl, 10 mM EDTA, 7 M urea) for 20 min at 37 °C. RNAs were extracted by Acid-Phenol: Chloroform, pH 4.5 (Ambion) and precipitated in pure ethanol with the help of glycogen (Thermo). The recovered RNAs were subjected to mononucleoside UHPLC-MRM-MS/MS analysis.

### *In vivo* RIP and RNA-BisSeq assay

The procedure was adapted from a previous report^4^ with some modifications. Flag-ALYREF overexpressed cells pellets were resuspended with 2 volume of lysis buffer (150 mM KCl, 10 mM HEPES pH 7.6, 2 mM EDTA, 0.5% NP-40, 0.5 mM DTT, 1:100 protease inhibitor cocktail, 400 U/ml RNase inhibitor), and incubated at 4 °C for 30 min with rotation. Then the lysate was centrifuged at 15 000 g for 20 min. The anti-Flag M2 magnetic beads (Sigma, 10 μl per mg lysate) were washed with a 600 ml NT2 buffer (200 mM NaCl, 50 mM HEPES pH 7.6, 2 mM EDTA, 0.05% NP-40, 0.5 mM DTT, 200 U/ml RNase inhibitor) four times and then resuspended in 800 ml ice-cold NT2 buffer. Cell lysate was mixed with M2 beads and incubated at 4 °C for 4 h with rotation. The beads were washed two times with 1 ml ice-cold NT2 buffer. Then the beads were subject to Micrococal nuclease (NEB) digestion (1:1 000 000 dilution) for 8 min at 37 °C. The beads were cooled on ice immediately for 5 min and washed two times with 1 ml ice-cold 1× PNK+EGTA buffer (50 mM Tris-HCl pH 7.5, 20 mM EDTA, 0.05% NP-40, 200 U/ml RNase inhibitor) and two times with 1 ml ice-cold 1× PK buffer (50 mM NaCl, 100 mM Tris-HCl pH 7.5, 10 mM EDTA, 0.2% SDS, 200 U/ml RNase inhibitor). Then the beads were digested with 200 μl pre-heated (20 min at 50 °C) Proteinase K solution (4 mg/ml) for 40 min at 50 °C with rotation at 2 000 rpm/min. After centrifugation at top speed for 5 min, the supernatant was transferred to a new 1.5 ml tube and RNAs extracted with an equal volume of Acid-Phenol: Chloroform, pH 4.5 (Ambion). The RNAs were subjected to RNA-BisSeq and RNA seq.

### EMSA

Purified wild-type Flag-tagged ALYREF and mutant proteins were diluted to a series of concentrations of 0.2 μM, 0.5 μM, 1 μM, and 2 μM in binding buffer (50 mM Tris-HCl pH 7.5, 100 mM NaCl, 0.4 mM EDTA, 0.1% NP-40, and 40 U/ml RNasin, 1 mM DTT, 50% glycerol, 5 ng/μl BSA). 1 μl synthesized RNA probe with or without m^5^C (100 nM final concentration) and 1 μl purified protein (10 nM, 50 nM, 100 nM, and 200 nM final concentration, respectively) were mixed and incubated at room temperature for 30 min. Then, 1 μl glutaraldehyde (0.2% final concentration) was added into the mixture which was incubated at room temperature for 15 min. The entire 11 μl RNA-protein mixture was mixed with 5 μl 5× Hi-Density TBE Sample buffer and separated on 6% TBE gel on ice for 30 min at 80 V. The gel was transferred onto positive charged nylon transfer membrane (GE Healthcare) and nucleic acids detected by the chemiluminescent nucleic acid detection module (Thermo) following the manufacturer's instructions. Quantification of each band was carried out using Quantity One software (Bio-Rad). The RNA binding ratio at each protein concentration was determined by (RNA-protein)/((free RNA) + (RNA-protein)).

### PAR-CLIP

HeLa cells with NSUN2 knockdown and ALYREF reconstitution, or ALYREF knockdown and NSUN2 reconstitution, were cultured in medium supplemented with 200 μM 4-thiouridine (4-SU) (Sigma) for 14 h, and then irradiated once with 400 mJ/cm^2^ at 365 nm using the CL-1000 Ultraviolet Crosslinker (UVP) for crosslinking. Cells were harvested in lysis buffer (50 mM Tris-HCl pH 7.5, 100 mM NaCl, 2 mM EDTA, 0.5% (v/v) NP-40, 1 mM NaF, 1× Protease Inhibitor Cocktail (Sigma), 0.04 U/ml RNasin (Promega)) and rotated for 30 min at 4 °C. Cell debris was removed by centrifugation at 14 000 rpm for 30 min at 4 °C and the supernatant (3-4 mg/ml) was digested by 1 U/μl RNase T1 at 22 °C in a water bath for 8 min and cooled on ice for 5 min. Then the lysates were incubated with anti-Flag M2 magnetic beads (Sigma) for 2 h at 4 °C and the immunoprecipitates were then washed three times with IP wash buffer (50 mM Tris-HCl pH 7.5, 300 mM NaCl, 0.05% (v/v) NP-40, 1× Protease Inhibitor Cocktail (Sigma), 0.04 U/ml RNasin (Promega)). Beads were digested with 10 U/μl RNase T1 again at 22 °C in a water bath for 8 min, cooled on ice for 5 min, then washed three times in high salt wash buffer (50 mM Tris-HCl pH 7.5, 500 mM NaCl, 0.05% (v/v) NP-40, 1× Protease inhibitor cocktail (Sigma), 0.04 U/ml RNasin (Promega)), resuspended in 100 μl dephosphorylation buffer (50 mM Tris-HCl pH 7.9, 100 mM NaCl, 10 mM MgCl_2_), and incubated with 0.5 U/μl calf intestinal alkaline phosphatase (CIP, NEB) for 10 min at 37 °C with gentle rotation. Beads were then washed twice with phosphatase wash buffer (50 mM Tris-HCl pH 7.5, 20 mM EGTA, 0.5% (v/v) Triton X-100) with 3 min rotation.

For PAR-CLIP-biotin chemiluminescent nucleic acid detection, the protein-RNA-beads complex was labeled with biotin using the RNA 3′ End Biotinylation kit (Thermo) following the manufacturer's instructions. After washing three times with IP wash buffer, beads were resuspended in 20 μl 2× LDS loading buffer (Invitrogen) and 40 μl 1× LDS loading buffer (Invitrogen), boiled at 95 °C for 10 min. To detect RNA-protein complexes, one sixth of the samples were separated by SDS-PAGE and visualized by the chemiluminescent nucleic acid detection module (Thermo) following the manufacturer's instructions. One sixth of the samples were separated by SDS-PAGE to detect the immunoprecipitation efficiency. The relative density of RNA bound by specific protein was analyzed by Quantity One.

### Immunofluorescence

HeLa cells at around 30% confluence were transfected with 100 nM 5′-FAM labeled siRNA using RFect siRNA transfection reagent (BIO-TRAN). The cells were transfected again at 24 h post the first transfection. HeLa cells grown on the coverslips were rinsed twice with PBS and fixed with 4% paraformaldehyde in PBS for 10 min on ice. After washing three times with PBS, cells were permeabilized with 0.2% TritonX-100 in PBS for 10 min on ice. Cells were then washed twice with PBS, blocked with 5% milk in PBST for 30 min at 37 °C and incubated for 1 h at 37 °C with primary antibodies at the dilution ratio as indicated. After washing with PBST, cells were incubated with corresponding Cy3 conjugated anti-rabbit IgG (sigma, C2306) or FITC conjugated anti-mouse IgG (sigma, F2883) secondary antibody for 30 min at 37 °C. Coverslips were then mounted with DAPI-containing mounting medium (Vector Laboratories, H-1200). Fluorescent images were acquired using a Leica TCS SP8 confocal microscope. The relative mean fluorescence densities were analyzed by Image-Pro Plus, and plotted using GraphPad Prism 6 software.

For co-localization analysis, cells grown on coverslips were incubated with a mixture of different sources of (rabbit or mouse) primary antibodies followed by incubation with a mixture of Cy3 conjugated anti-rabbit IgG and FITC conjugated anti-mouse IgG secondary antibodies. The changes in intensities of co-localization signals were shown by the line scan graph of fluorescence intensity using LAS AF Lite (Leica Microsystems) as described in previous studies^14,59,60^.

### RNA *in situ* hybridization

HeLa cells grown on coverslips were fixed with 4% formaldehyde in PBS at room temperature for 20 min. Cells were then permeabilized with 0.5% TritonX-100 in PBS for 15 min, and washed twice with PBS. Hybridization was performed for 5 h at 37 °C in the mixture containing 20% formamide, 2× SSC, 1 mg/ml tRNA (Sigma), 10% dextransulfate. Cy3 labeled oligo(dT)_50_ probes (Sangon Biotech) were used for analyzing endogenous mRNA export. Cy3-labeled oligonucleotide probes complementary to *EGFP* mRNAs were used for analyzing nuclear export of *EGFP-FBXW9* Exon 1 minigene. After washing three times with 2× SSC buffer and once with 1× SSC buffer, coverslips were then mounted with DAPI-containing mounting medium (Vector Laboratories). Optical sections were captured with a Leica TCS SP8 confocal microscope. The probes are listed in Supplementary information, Table S5.

### Isolation of cytoplasmic and nuclear RNAs for qPCR

Cytoplasmic and nuclear RNA isolation was performed using the Ambion PARIS Protein and RNA Isolation System (Ambion) according to the manufacturer's instructions. The nuclear and cytoplasmic RNA fractions were analyzed by PCR using specific markers, 45S pre-rRNA for nucleus and RPS14 for cytoplasm. 5 μg nuclear RNA and the corresponding same volume of cytoplasmic RNA were used for cDNA synthesis using RevertAid^TM^ First Strand cDNA Synthesis Kit (Thermo).

For the qPCR-based mRNA export analysis, all the reactions were performed with Takara SYBR Premix Ex Taq (Takara) according to the manufacturer's instructions and quantified by a CFX96 Real-Time PCR System (Bio-Rad). The relative fold changes in cytoplasmic/nuclear ratios were calculated using the 2(-Delta Delta C(T)) method^61^. The primer pairs used for semi-quantitative PCR and qPCR in this study are listed in Supplementary information, Table S5.

### RNA-BisSeq bioinformatics analyses

Raw RNA-BisSeq reads for each sample were stripped of adaptor sequences and removed low quality bases using Trimmomatic^62^. The processed reads with lengths greater than 35 nt were defined as clean reads. Human and mouse reference genomes (version hg19 and mm10) were downloaded from UCSC database. The alignment procedure was performed by mapping the clean reads against hg19 or mm10 genome by Bismark (version 0.13.0)^63^ with stringent parameters: -N 0 -X 500. The unmapped reads were mapped against the transcriptome by Bismark with same parameters. The remaining reads were further mapped to the library collecting all exon-exon junctions based on the Ensembl annotation.

m^5^C sites were called by Bismark. To ensure the sufficient conversion efficiency, reads with > 30% unconverted cytosines that may reflecting insufficient bisulfite conversion were eliminated^24,29^. For the m^5^C sites in transcriptome and junction sequences, a custom script was used to convert them to corresponding genome locus to get the overall information of each m^5^C site. The methylation level is estimated as i/(i + j) where i represents number of reads showing methylation (C) at each m^5^C site, and j represents the number of reads lack of methylation (T). Only sites with coverage depth ≥ 30, methylation level ≥ 0.1 and methylated cytosine depth ≥ 5 were considered credible, for it is highly probable that the majority of sites with < 0.1 methylation level represent artifacts from various sources^24^. Each sample contains two replicates and only overlapped m^5^C sites between two replicates were used to the following analyses. This standard was also applied to all the following RNA-BisSeq data sets. For pseudouridylation^16^ and editing^31^ sites, only sites with modification or editing level ≥ 0.1 were used in the level analyses.

### Distribution of m^5^C sites

The m^5^C sites were annotated by applying BEDTools' intersectBed^64^. m^5^C sites located in mRNAs were mapped to four regions: CDSs, introns, 5′ UTRs and 3′ UTRs. If the site fell within a gene exon, then its position within the mature transcript was calculated using the exon lengths. This was then converted to a position within the 5′ UTR, the coding sequence, or the 3′ UTR segments, and divided by the length of that region and multiplied by 100 to determine a percentile for where this m^5^C fell. The percentile bin that the m^5^C fell into was then incremented, and the bins were plotted as a percentage of the total number of m^5^C sites in the data set. Then, the same was performed on m^6^A peak^3^ and total “C” along mRNA transcripts. The mRNA m^5^C sites were subdivided into three sequence contexts: CG, CHG, and CHH (H = A, C, U). To calculate the relative fractions of mRNA m^5^C sites in the three contexts, proportions of the three contexts in transcriptomes were calculated and the absolute m^5^C numbers in the three contexts were normalized to the proportions. To acquire the sequence preference proximal to m^5^C sites, 21 nt sequences centered with each m^5^C site were extracted with Bedtools, and logo plots were generated with WebLogo^65^. Gene ontology (GO) analysis of m^5^C-containing mRNAs was performed using the DAVID bioinformatics database^66^. Only GO terms of the genes in biological process category are shown. GO terms with *P* value of less than 0.05 were considered as statistically signiﬁcant.

### RNA-Seq analysis

For each sample with RNA-BisSeq, its transcriptome was also sequenced. The raw reads filtered by RNA-BisSeq data analysis method were mapped against the human genome (hg19) or mouse genome (mm10) references with TopHat2 (version 2.0.9)^67^. Two mismatches at the maximum were allowed and only uniquely mapped reads with mapping quality larger than or equal to 20 were kept for the subsequent analysis for each sample. The number of reads mapped to each Ensembl gene was counted using the HTSeq python package^68^, with the “union” overlap resolution mode. For each sample, RPKM was computed as the number of reads which map per kilobase of exon model per million mapped reads for each gene. mRNAs with RPKM ≥ 1 were regarded as expressing.

### Tissue specificity and dynamics of m^5^C methylation

Hierarchical clustering of m^5^C levels in six mouse tissues were determined in the R programming environment. Gene ontology (GO) analysis of expressed m^5^C-containing mRNAs (RPKM ≥ 1) in each tissue was performed by DAVID using expressed mRNAs (RPKM ≥ 1) as background. The m^5^C sites only occurring in one specific tissue were designated as tissue-specific RNA m^5^C sites. Considering the mRNA abundance may influence the identification of m^5^C sites, we first chose the mRNAs commonly expressed in all six tissues. The m^5^C sites occurring within the above mRNAs specifically in one tissue were regarded as stringent tissue-specific m^5^C sites.

For m^5^C sites in 3- and 4-week stage testis samples, only the sites within mRNAs expressed (RPKM ≥ 1) in both stages were used in the following analyses. The m^5^C sites only occurring in 3- or 4-week stage testis were defined as 3- or 4-week specific m^5^C sites, respectively. The difference in m^5^C site level that is greater than 5% between two stages was considered as increased or decreased m^5^C site methylations. Among m^5^C sites identified in control HeLa cells, sites with reduced methylation level > 5% in siNSUN2 samples were regarded as NSUN2-regulated sites.

### Identification of ALYREF target m^5^C sites

For RNA-BisSeq of ALYREF-RIP samples, m^5^C sites were identified with the same methods as mentioned above. For ALYREF-RIP-seq, the ALYREF binding regions (peaks) were identified using the MACS2 (version 2.0.10)^69^. The cutoff threshold for *P* value < 10^−5^ was set. To identify high-conﬁdence ALYREF target m^5^C sites, only m^5^C sites within ALYREF peaks were used in the analyses.

### Statistical analysis

All bioinformatics-associated statistical analyses (unless stated otherwise) were performed using the R package for statistical computing. For experimental quantification, the unpaired *t*-test of GraphPad Prism 6 software was applied and error bar was shown based on standard error of mean (SEM) (unless stated otherwise). *P* < 0.05 is considered as statistically significant.

### Statistical analysis and reproducibility

No statistical methods were used to predetermine sample size. The experiments were not randomized and the investigators were not blinded to allocation during experiments and outcome assessment.

### Accession number

The raw sequence data reported in this paper have been deposited in the Genome Sequence Archive^70^ in BIG Data Center^71^, Beijing Institute of Genomics (BIG), Chinese Academy of Sciences, under accession number PRJCA000315 that are publicly accessible at http://bigd.big.ac.cn/gsa, and also deposited in the Gene Expression Omnibus (GEO) under the accession number: GSE93751.

## Author Contributions

Y-G Y, Y-P S and H-L W conceived this project and supervised all the experiments. Y-G Y, YY, B-F S, Y-L Z, H-L W and Y-P S analyzed the data and wrote the manuscript. YY, XY and J-W X performed RNA-BisSeq. B-F S and Y-S C performed bioinformatics analysis with prediction and experimental candidate selection with assistance from H-Y S and QZ. XY, AL, W-Y L, and XW performed molecular biology, protein chemistry, and cell culture experiments with assistance from WX, D-P B, H-L M, SA, Y-J H, MS, BZ, C-M H, NH, G-B J, and Y-L Z.

## Competing Financial Interests

The authors declare no competing financial interests.

## Supplementary Information

Supplementary information, Figure S1 Transcriptome-wide identification of m^5^C sites by RNA-BisSeq.

Supplementary information, Figure S2 Conserved, tissue-specific and dynamic features of mRNA m^5^C methylation across mammalian transcriptomes.

Supplementary information, Figure S3 NSUN2 catalyzes the formation of m^5^C in mRNAs.

Supplementary information, Figure S4 ALYREF is an m^5^C-specific RNA binding protein.

Supplementary information, Figure S5 m^5^C regulates nuclear-cytoplasmic shuttling and RNA binding affinity of ALYREF.

Supplementary information, Figure S6 m^5^C participates in mRNA export regulation.

Supplementary information, Figure S7 ALYREF mediated m^5^C regulates mRNA export.

Supplementary information, Table S1 Summary of information on sequencing samples.

Supplementary information, Table S2 Dataset of all identified m^5^C sites in normal or NSUN2 deficient human HeLa cells.

Supplementary information, Table S3 Dataset of all identified m^5^C sites in six mouse tissues (small intestine, heart, muscle, brain, kidney and liver), 3 and 4 week mouse testis samples.

Supplementary information, Table S4 Dataset of all identified m^5^C sites and ALYREF peaks in ALYREF-RIP samples.

Supplementary information, Table S5 Information of primer and siRNA sequences used in this study.
