# Supplementary material for: 5-methylcytosine promotes mRNA export — NSUN2 as the methyltransferase and ALYREF as an m5C reader
Source: Cell Res. 2017 Apr 18;27(5):606–25. doi: 10.1038/cr.2017.55 (PMC5594206; doi:10.1038/cr.2017.55)
Supplement: Supplementary information, Figure S1 — Transcriptome-wide identification of m5C sites by RNA-BisSeq. [file cr201755x1.pdf]

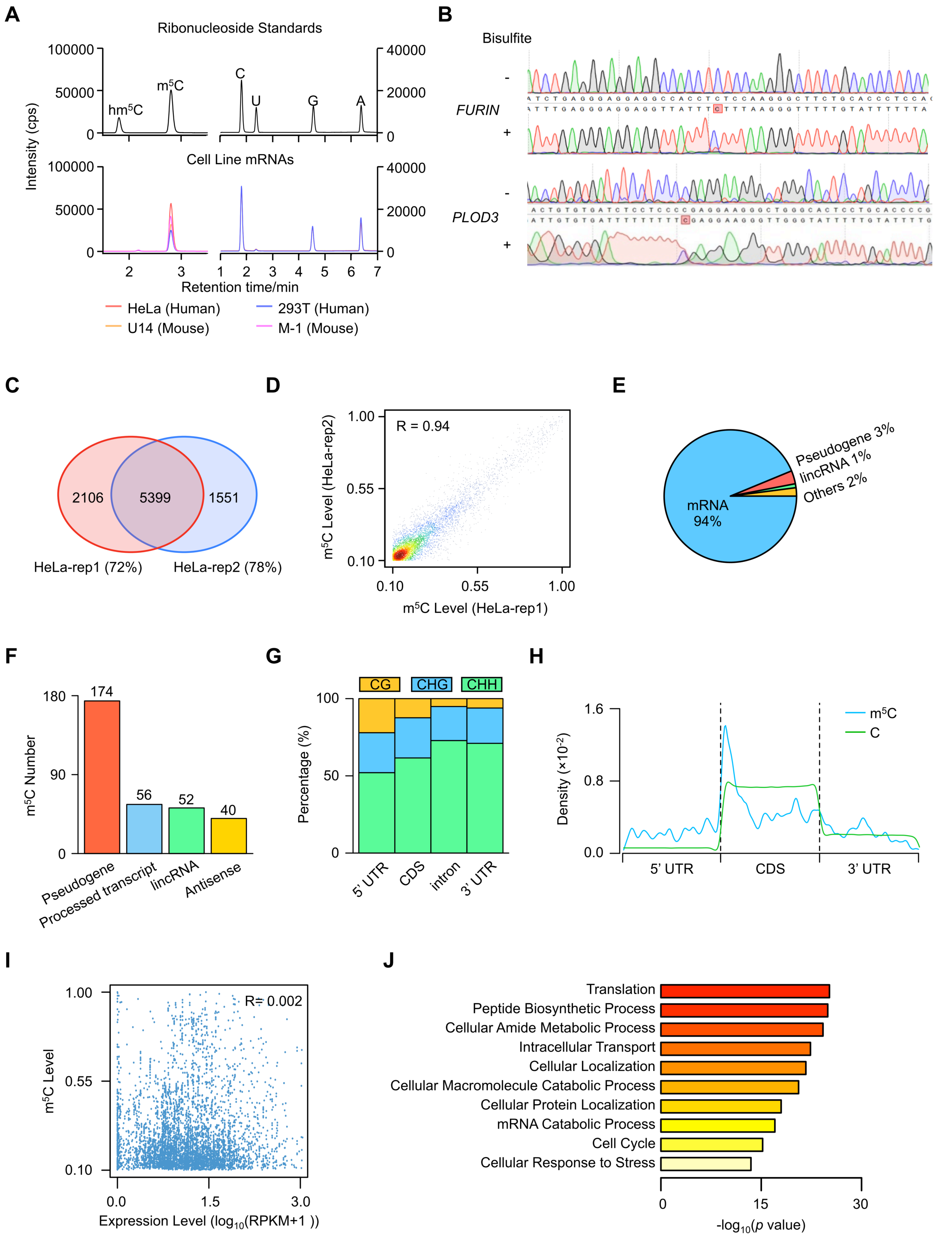

Supplementary information, Figure S1 Transcriptome-wide identification of m^5C sites by RNA-BisSeq. **(A)** For ribonucleoside standards, 5 fmol of each of m^5C and hm^5C, 20 fmol of each of C, G, and A, 400 fmol of U were used. 40 ng of mRNA for each sample was used to

analyze m^5C and hm^5C, and 0.02 ng was used to analyze C, U, G and A. **(B)** m^5C sites within *FURIN* (chr15:91425541) and *PLOD3* (chr7:100849671) (hg19) identified by RNA-BisSeq were validated. cDNA was amplified by PCR using normal primers for untreated mRNAs and specific primers for bisulfite treated mRNAs. **(C)** Venn diagram showing the overlap of m^5C sites within RNAs between two HeLa replicates. **(D)** The Pearson correlation of common m^5C levels between two HeLa replicates. **(E)** Percentage of m^5C sites within various RNA categories. **(F)** Bar chart showing numbers of m^5C sites within representative noncoding RNAs. **(G)** Bar chart showing the percentage of CG, CHG and CHH contexts within 5' UTR, CDS, intron and 3' UTR regions in transcriptomes. **(H)** Distribution of m^5C sites and C sites along mRNA transcripts. The moving averages of percentages of mRNA m^5C and C sites were shown. **(I)** Relationship between m^5C levels and mRNA abundance in HeLa cells (R = 0.002). **(J)** Representative gene ontology (GO) terms enriched in m^5C-containing mRNAs.
