# Supplementary material for: 5-methylcytosine promotes mRNA export — NSUN2 as the methyltransferase and ALYREF as an m5C reader
Source: Cell Res. 2017 Apr 18;27(5):606–25. doi: 10.1038/cr.2017.55 (PMC5594206; doi:10.1038/cr.2017.55)
Supplement: Supplementary information, Figure S2 — Conserved, tissue-specific and dynamic features of mRNA m5C methylation across mammalian transcriptomes. [file cr201755x2.pdf]

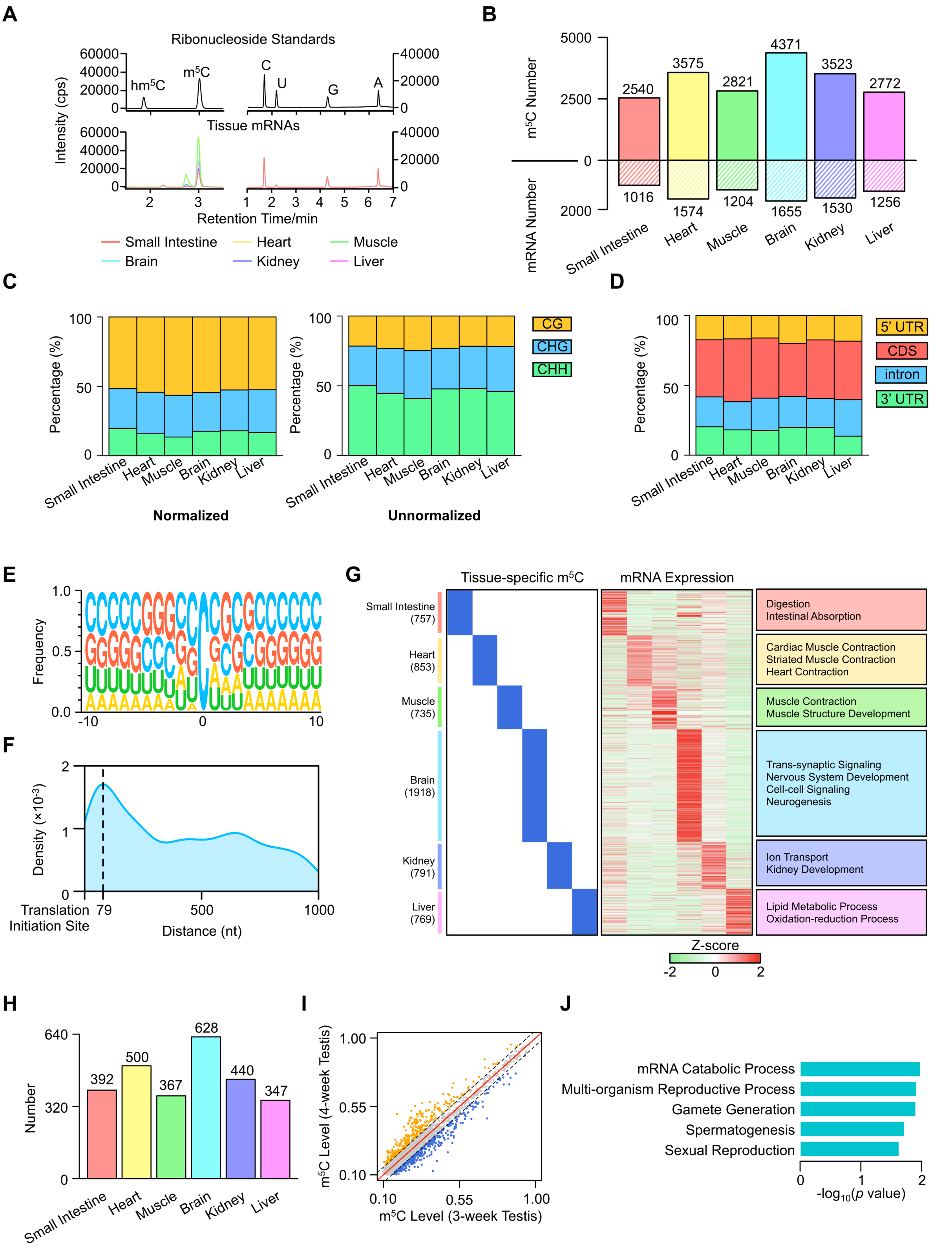

Supplementary information, Figure S2 Conserved, tissue-specific and dynamic features of mRNA m^{5}C methylation across mammalian transcriptomes. **(A)** UHPLC-MRM-MS/MS analysis of m^{5}C and hm^{5}C in mRNAs from six mouse tissues (small intestine, heart, muscle, brain, kidney and liver). The same ribonucleoside standards and mRNA amount as cell lines

were used. **(B)** Bar charts showing numbers of m^5C sites and m^5C-containing mRNAs in six mouse tissues. **(C)** Bar charts showing the normalized (left panel) and unnormalized (right panel) fractions of mRNA m^5C sites identified in each sequence context: CG, CHG and CHH, where H = A, C, or U in six mouse tissues. **(D)** Bar charts showing the percentage of m^5C sites within distinct RNA regions: CDS, intron, 5' UTR and 3' UTR among six mouse tissues. **(E)** Sequence frequency logo for the sequences proximal to mRNA m^5C sites in mouse tissues. **(F)** Distribution of m^5C sites (pooled from mouse tissues) in CDS regions of mRNA transcripts. The distance of m^5C sites in mRNA transcripts to translation initiation sites was determined and plotted. There was a peak of m^5C number at around 79 nt after translation initiation sites. **(G)** Heatmap showing tissue-specific m^5C sites and expression levels of mRNAs containing them. Blue lines represent presence of tissue-specific m^5C sites. The representative GO term enrichment in mRNA with tissue-specific sites in each tissue was shown. **(H)** Bar charts showing numbers of tissue-specific m^5C sites within mRNAs expressed in all six tissues. **(I)** Scatter plot showing the levels of common m^5C sites between 3- and 4-week testis samples. The m^5C sites with increased or decreased methylation levels in 4-week testis compared to 3-week testis were highlighted with orange or blue color, respectively. **(J)** Gene ontology analysis of mRNAs containing m^5C sites with differential levels in 4-week stage testis.
