# Supplementary material for: 5-methylcytosine promotes mRNA export — NSUN2 as the methyltransferase and ALYREF as an m5C reader
Source: Cell Res. 2017 Apr 18;27(5):606–25. doi: 10.1038/cr.2017.55 (PMC5594206; doi:10.1038/cr.2017.55)
Supplement: Supplementary information, Figure S3 — NSUN2 catalyzes the formation of m5C in mRNAs. [file cr201755x3.pdf]

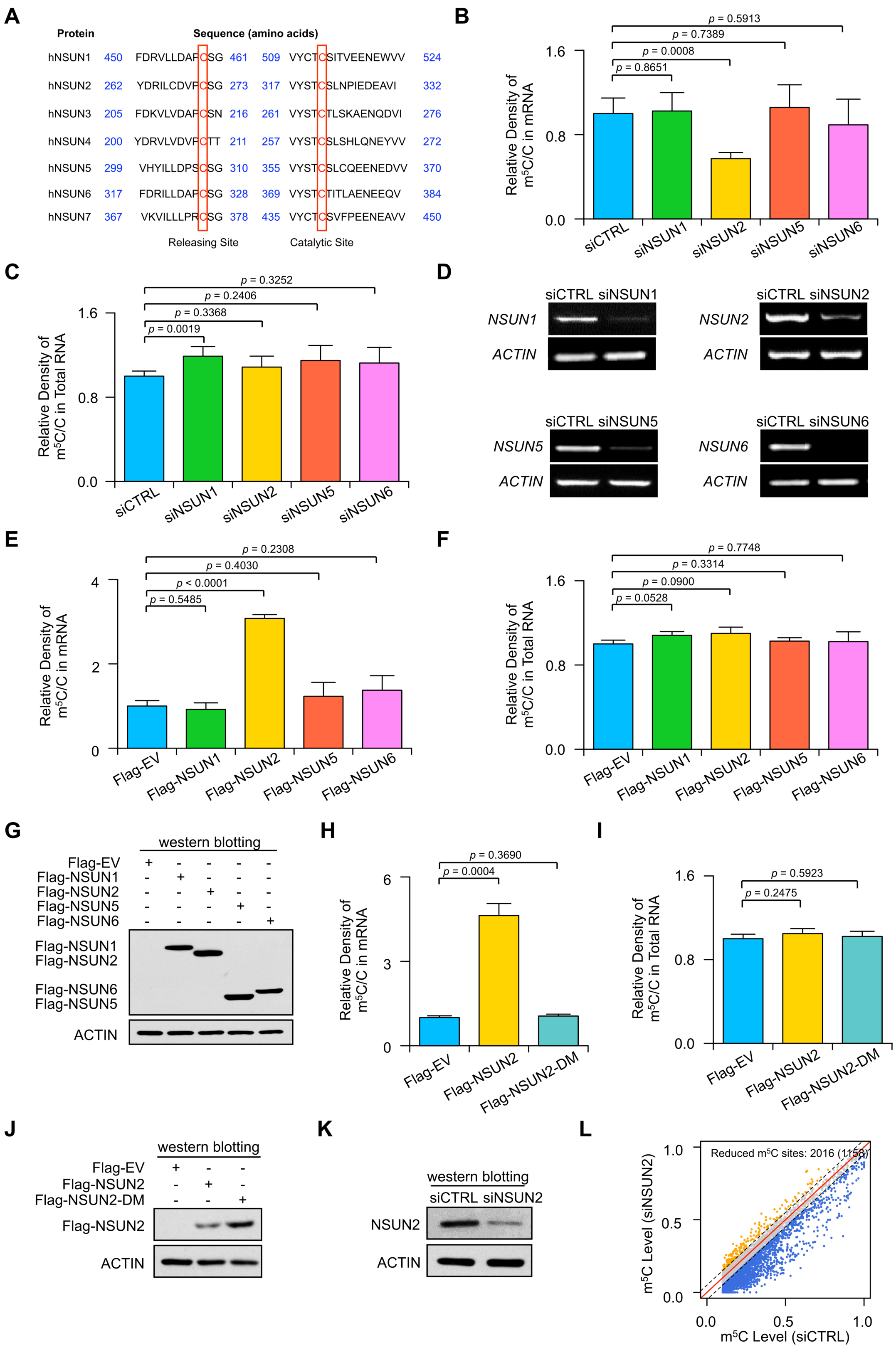

Supplementary information, Figure S3 NSUN2 catalyzes the formation of m^5C in mRNAs.

(A) Multiple sequence alignments of the NOL1/NOP2/SUN domain family members

(Genebank: NP_001028886.1, NP_060225.4, NP_071355.1, NP_001243056.1, NP_683759.1, NP_872349.1, NP_078953.3). Red boxes: conversed cytosine sites necessary for methyltransferase activity. **(B, C)** UHPLC-MRM-MS/MS analysis of m^5C levels within mRNAs **(B)** or total RNAs **(C)** upon individual knockdown of NSUN1, NSUN2, NSUN5 or NSUN6. **(D)** Validation of knockdown efficiency by semi-quantitative RT-PCR. ACTIN was used as loading control. **(E, F)** UHPLC-MRM-MS/MS analysis of m^5C levels within mRNAs **(E)** or total RNAs **(F)** upon individual overexpression of NSUN1, NSUN2, NSUN5 or NSUN6. **(G)** Expression level validated by western blotting using anti-Flag antibody. ACTIN was used as loading control. **(H, I)** UHPLC-MRM-MS/MS analysis of m^5C levels within mRNAs **(H)** or total RNAs **(I)** upon individual overexpression of Flag-NSUN2 or Flag-NSUN2-DM (C271A/C321A). **(J)** Validation of Flag-NSUN2 or Flag-NSUN2-DM (C271A/C321A) expression by western blotting. ACTIN was used as loading control. For UHPLC-MRM-MS/MS, 40 ng of total RNA was used to analyze m^5C, and 0.02 ng was used to analyze C, U, G, and A. *p* values, Student's *t*-test. Error bars, mean ± SEM (n = 3). **(K)** Validation of knockdown efficiency of NSUN2 in HeLa cells by western blotting using anti-NSUN2 antibody. ACTIN was used as loading control. **(L)** Scatter plot showing m^5C levels in siCTRL and siNSUN2 samples. To m^5C sites in control HeLa cells, sites with increased or reduced methylation level upon NSUN2 knockdown were highlighted with orange or blue color, respectively. 2,016 m^5C sites (within 1,158 mRNAs) present in control HeLa cells were with reduced methylation levels upon NSUN2 knockdown.
