# Supplementary material for: 5-methylcytosine promotes mRNA export — NSUN2 as the methyltransferase and ALYREF as an m5C reader
Source: Cell Res. 2017 Apr 18;27(5):606–25. doi: 10.1038/cr.2017.55 (PMC5594206; doi:10.1038/cr.2017.55)
Supplement: Supplementary information, Figure S4 — ALYREF is an m5C-specific RNA binding protein. [file cr201755x4.pdf]

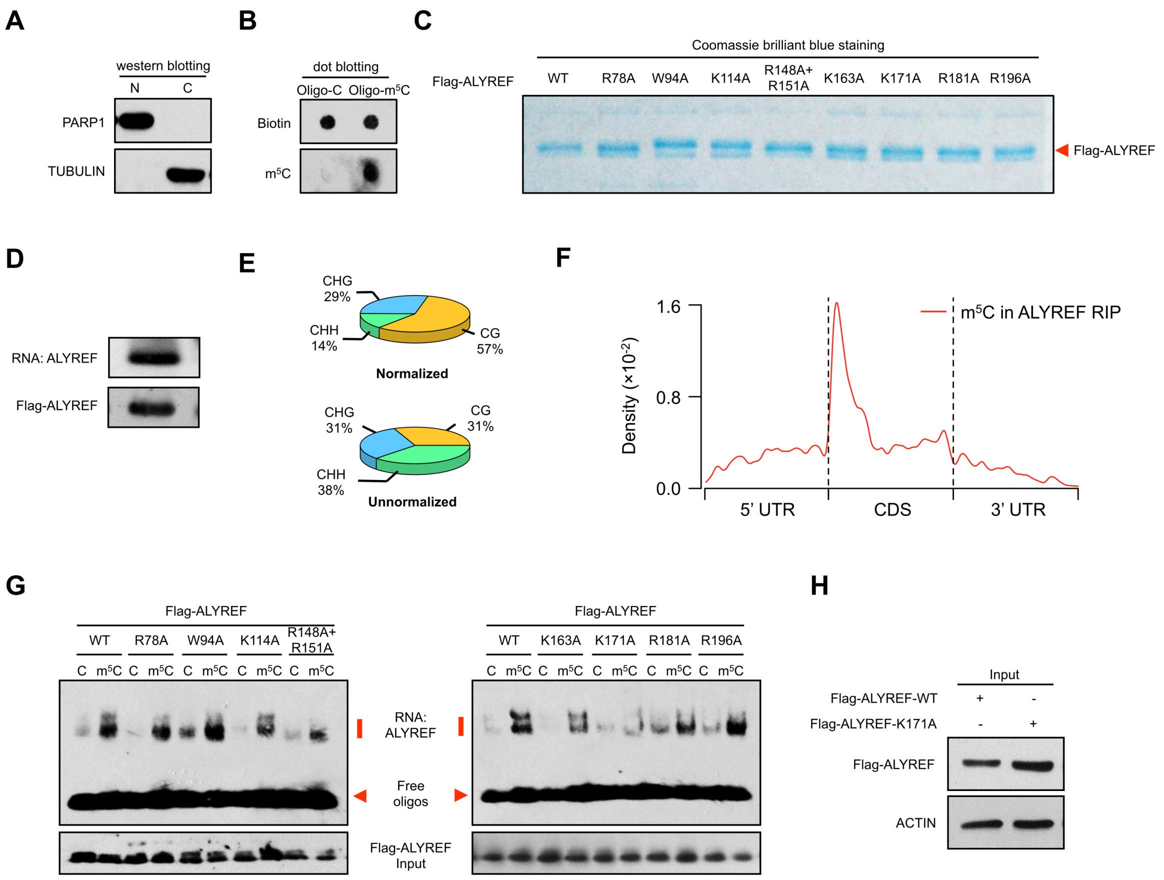

Supplementary information, Figure S4 ALYREF is an m^{5}C-specific RNA binding protein. **(A)** Isolation of the nuclear and cytoplasmic fractions from HeLa cells detected by western blotting. PARP1: nuclear marker; TUBULIN: cytoplasmic marker. **(B)** Detection of m^{5}C in synthesized RNA oligos with m^{5}C (Oligo-m^{5}C) or without m^{5}C (Oligo-C) modification by dot blotting assay. Biotin was used as equal loading control. **(C)** Coomassie brilliant blue staining of purified full length wild-type Flag-ALYREF (WT) and mutants (R78A, W94A, K114A, R148A& R151A, K163A, K171A, R181A, R196A). **(D)** Chemiluminescent detection of biotin-labeled mRNAs in ALYREF-RIP sample. ALYREF-RIP efficiency was validated by western blotting using anti-Flag antibody (bottom). **(E)** The normalized and unnormalized proportions of m^{5}C sites in in vivo ALYREF-RIP mRNA samples identified in each sequence context: CG, CHG and CHH, where H = A, C, or U. **(F)** Distribution of ALYREF target m^{5}C sites along mRNA transcripts in *in vivo* ALYREF-RIP samples. **(G)** EMSA showing the RNA binding ability of Flag-ALYREF wild-type (WT) and mutants (R78A, W94A, K114A, R148A&R151A, K163A, K171A, R181A, R196A) with Oligo-m^{5}C

or Oligo-C. 100 nM RNA oligos and 200 nM proteins were used. **(H)** Western blotting validation of Flag-ALYREF-WT and Flag-ALYREF-K171A expression in HeLa cells. ACTIN was used as loading control.
