# Supplementary material for: 5-methylcytosine promotes mRNA export — NSUN2 as the methyltransferase and ALYREF as an m5C reader
Source: Cell Res. 2017 Apr 18;27(5):606–25. doi: 10.1038/cr.2017.55 (PMC5594206; doi:10.1038/cr.2017.55)
Supplement: Supplementary information, Figure S5 — m5C regulates nuclear-cytoplasmic shuttling and RNA binding affinity of ALYREF. [file cr201755x5.pdf]

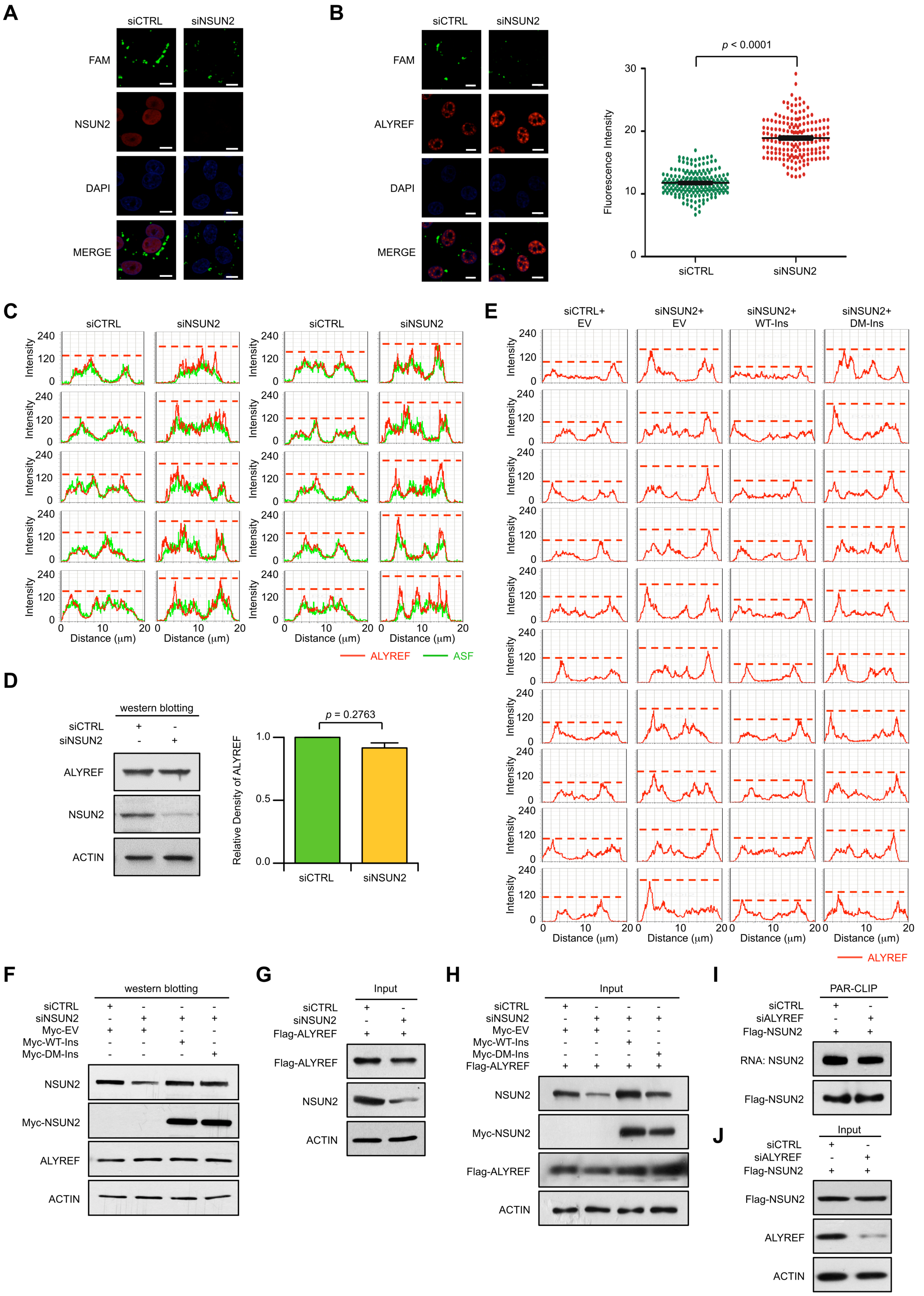

Supplementary information, Figure S5 m^{5}C regulates nuclear-cytoplasmic shuttling and RNA binding affinity of ALYREF. **(A)** Knockdown efficiency of NSUN2 with FAM-labeled

siRNAs detected by immunofluorescence staining. Green: FAM-labeled siRNAs; Red: siRNA targeted proteins. Scale bar, 10 μm. **(B)** Immunofluorescence staining of ALYREF in nuclear speckle (red) upon NSUN2 knockdown with FAM-labeled siRNAs (green) (left panel) and quantification of ALYREF staining signal (right panel). Scale bar, 10 μm. *p* values, Student's *t*-test. Error bars, mean ± SEM (*n* = 120). **(C)** Representative line scan graphs of ALYREF (red) and ASF (green) fluorescence intensities in the control or NSUN2 knockdown HeLa cells. ASF serves as nuclear speckle marker. **(D)** Western blotting (left panel) and quantification (right panel) of the total protein abundance of ALYREF in control or NSUN2 knockdown HeLa cells. ACTIN serves as loading control. *p* values, Student's *t*-test. Error bars, mean ± SEM (*n* = 3). **(E)** Representative line scan graphs of ALYREF (red) fluorescence intensities in NSUN2 knockdown HeLa cells reconstituted with control EGFP (EGFP-EV), EGFP-tagged siNSUN2-insensitive wild-type NSUN2 (EGFP-WT-Ins) or mutant (EGFP-DM-Ins). **(F)** Levels of NSUN2, Myc-WT-Ins and Myc-DM-Ins protein examined by western blotting. ACTIN was used as loading control. **(G)** NSUN2 knockdown efficiency and Flag-ALYREF overexpression in NSUN2 knockdown HeLa cells examined by western blotting. ACTIN was used as loading control. **(H)** Levels of NSUN2, Flag-ALYREF, siNSUN2-insensitive Myc-NSUN2 wild-type (Myc-WT-Ins) and double-mutant (Myc-DM-Ins) in control and NSUN2 knockdown HeLa cells used for PAR-CLIP examined by western blotting. ACTIN was used as loading control. **(I)** PAR-CLIP analysis of RNAs pull-downed by Flag-NSUN2 in control or ALYREF knockdown HeLa cells. RNA labeled with biotin at 3' end of RNA (End Biotinylation Kit, Thermo) was visualized by the Chemiluminescent nucleic acid detection module. Flag-NSUN2 IP efficiency was examined by western blotting using anti-Flag antibody (bottom). **(J)** ALYREF knockdown efficiency and Flag-NSUN2 overexpression in ALYREF knockdown HeLa cells examined by western blotting. ACTIN was used as loading control.
