# Supplementary material for: 5-methylcytosine promotes mRNA export — NSUN2 as the methyltransferase and ALYREF as an m5C reader
Source: Cell Res. 2017 Apr 18;27(5):606–25. doi: 10.1038/cr.2017.55 (PMC5594206; doi:10.1038/cr.2017.55)
Supplement: Supplementary information, Figure S6 — m5C participates in mRNA export regulation. [file cr201755x6.pdf]

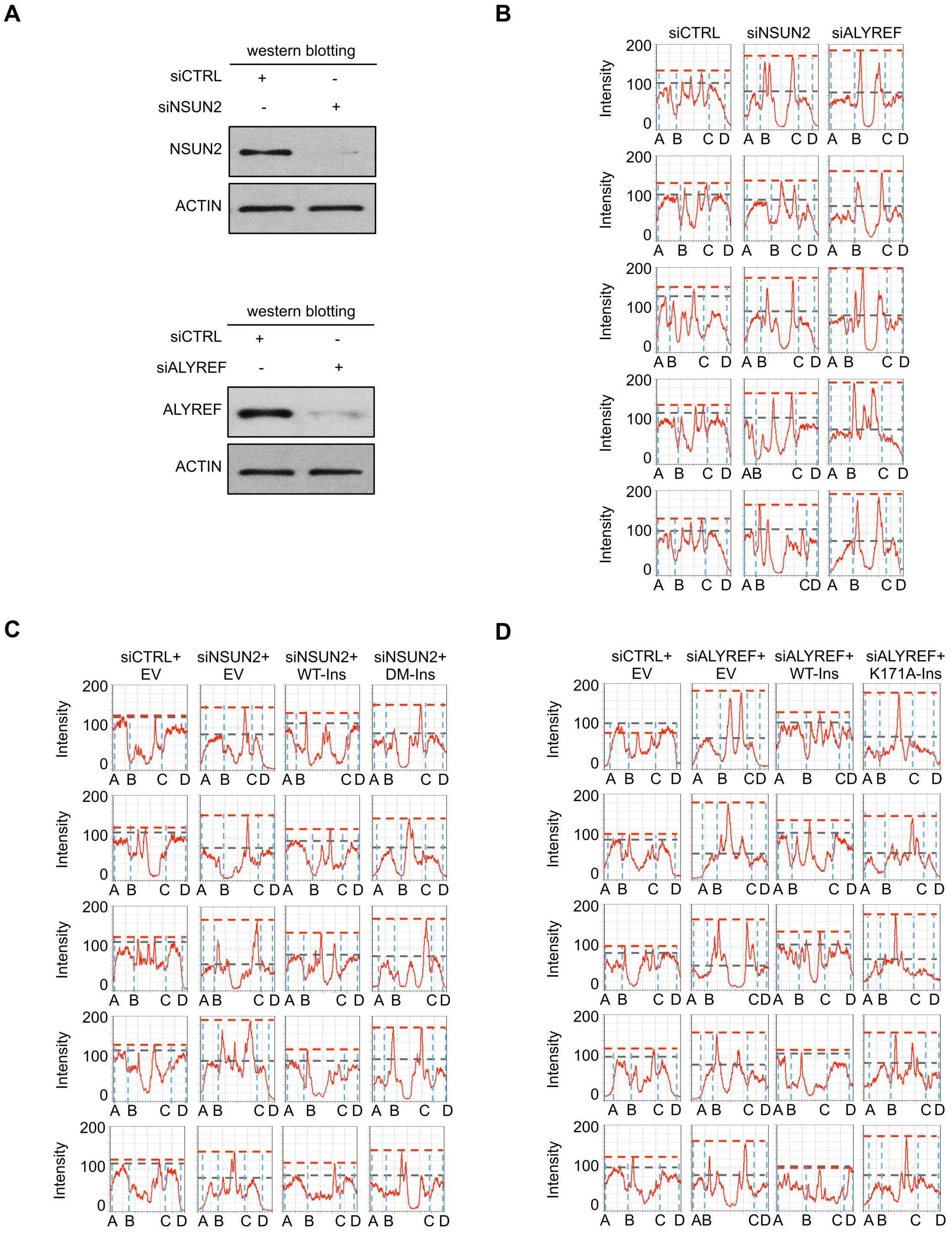

Supplementary information, Figure S6 m^{5}C participates in mRNA export regulation. **(A)** Western blotting validation of knockdown efficiency of NSUN2 (top) and ALYREF (bottom). ACTIN was used as loading control. **(B)** Representative line scan graphs of mRNA fluorescence intensities in the control, NSUN2 or ALYREF knockdown HeLa cells. The red and black lines represent the peak densities of nuclear and cytoplasmic mRNAs, respectively.

A-B and C-D: cytoplasmic regions; B-C: nuclear region. **(C, D)** Representative line scan graphs of mRNA fluorescence intensities in NSUN2 **(C)** or ALYREF **(D)** knockdown HeLa cells reconstituted with control vector, EGFP/GFP-tagged wild-type or NSUN2 (**C**)/ALYREF **(D)** mutant. The red and black lines represent the peak densities of nuclear and cytoplasmic mRNAs, respectively. A-B and C-D: cytoplasmic regions; B-C: nuclear region.
