# Supplementary material for: 5-methylcytosine promotes mRNA export — NSUN2 as the methyltransferase and ALYREF as an m5C reader
Source: Cell Res. 2017 Apr 18;27(5):606–25. doi: 10.1038/cr.2017.55 (PMC5594206; doi:10.1038/cr.2017.55)
Supplement: Supplementary information, Figure S7 — ALYREF mediated m5C regulates mRNA export. [file cr201755x7.pdf]

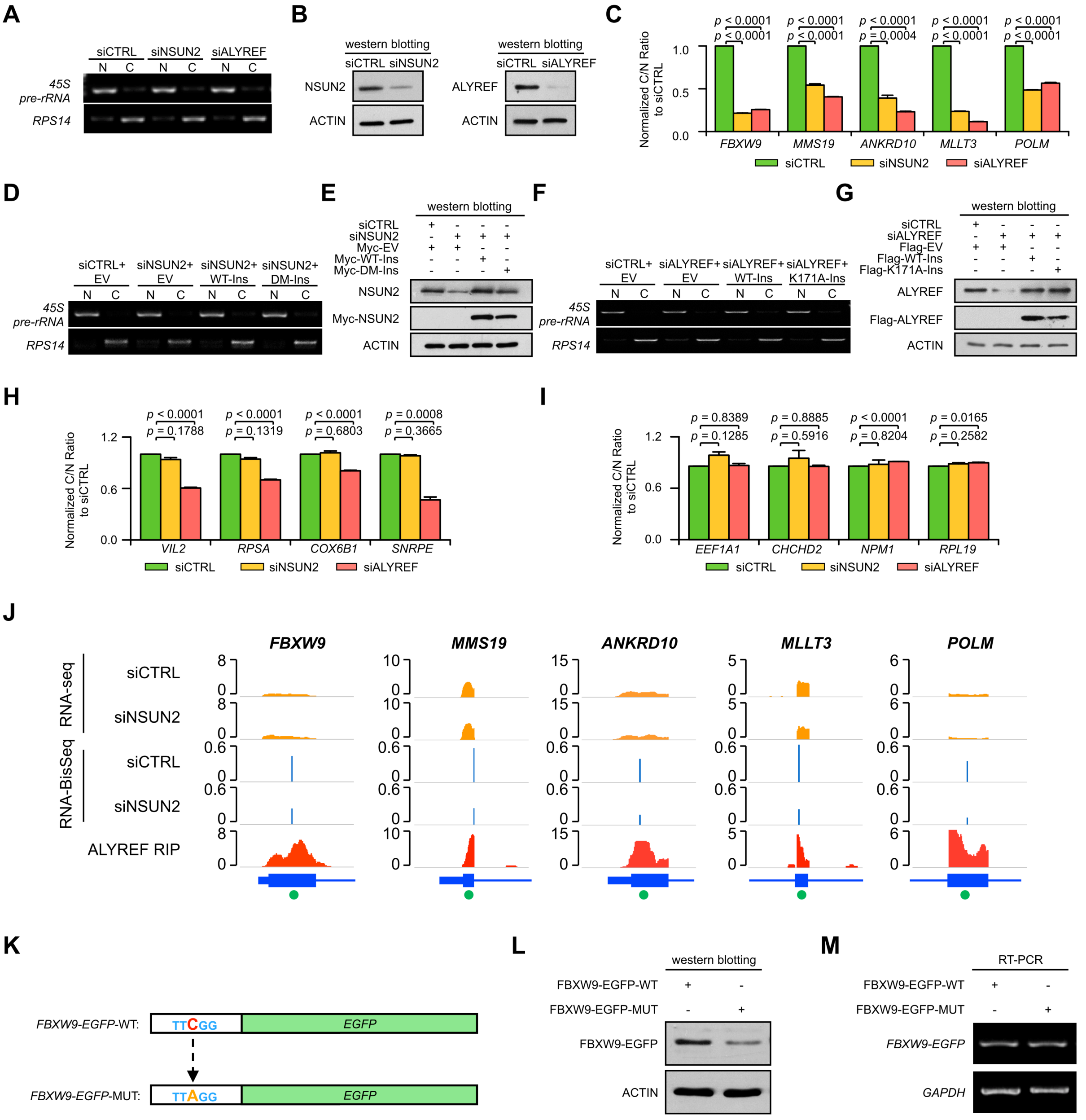

Supplementary information, Figure S7 ALYREF mediated m^{5}C regulates mRNA export. **(A, B)** RT-PCR validation of the isolated cytoplasmic and nuclear RNA fractions **(A)** and western blotting analysis of knockdown efficiency of NSUN2 or ALYREF **(B)** in the control, NSUN2 or ALYREF knockdown HeLa cells. *45S pre-RNA* and *RPS14* RNA serve as nuclear and cytoplasmic RNA markers, respectively. ACTIN was used as protein loading control. **(C)** qPCR analysis of the relative cytoplasmic to nuclear mRNA ratios (C/N) of NSUN2 target genes with m^{5}C modification in the control, NSUN2 or ALYREF deficient cells. **(D, E)** RT-PCR validation of the isolated cytoplasmic and nuclear RNA fractions **(D)** and western

blotting **(E)** analysis of levels of NSUN2, siNSUN2-insensitive Myc-NSUN2 wild-type (Myc-WT-Ins) and double-mutant (Myc-DM-Ins) in control and NSUN2 knockdown HeLa cells used for qPCR. **(F, G)** RT-PCR validation of the isolated cytoplasmic and nuclear RNA fractions **(F)** and western blotting **(G)** analysis levels of ALYREF, siALYREF-insensitive wild-type Flag-ALYREF (Flag-WT-Ins) and its m^5C binding defective mutant (Flag-K171A-Ins) in control and ALYREF knockdown HeLa cells. **(H, I)** qPCR analysis of the relative cytoplasmic to nuclear mRNA ratios (C/N) of m^5C-modified genes untargeted by NSUN2 **(H)** and genes without m^5C modification **(I)** in the control, NSUN2 or ALYREF knockdown cells. **(J)** Browser representing m^5C levels, RNA-seq and ALYREF RIP-seq tracks of *FBXW9*, *MMS19*, *ANKRD10*, *MLLT3*, *POLM* in control and NSUN2 knockdown HeLa cells. The RNA-seq and ALYREF RIP-seq tracks represent read counts per million mapped reads (RPM). ALYREF peaks were labeled with green dots. **(K)** Schematic representation of pEGFP-*FBXW9* minigene construct containing *FBXW9* Exon 1 with one m^5C site (cytosine 215 from start codon AUG) (*FBXW9*-WT). Cytosine was mutated to Adenine to generate *FBXW9*-MUT construct. **(L, M)** Protein **(L)** and mRNA **(M)** levels of FBXW9-EGFP-WT and FBXW9-EGFP-MUT in HeLa cells. ACTIN and GAPDH serve as controls. *p* values, Student's *t*-test. Error bars, mean ± SEM (n = 3).
